# Small-Scale High-Fidelity Simulation for Mass Casualty Incident Readiness

**DOI:** 10.21980/J84S8S

**Published:** 2021-10-15

**Authors:** Seanne Facho, Andrea Weiers, Amber Jones, Sage Wexner, Jessie Nelson

**Affiliations:** * Kern Medical Center, Department of Emergency Medicine, Bakersfield, CA; ^ Regions Hospital, Department of Emergency Medicine, St. Paul, MN

## Abstract

**Audience:**

This content can be used for trauma centers, emergency medicine residency programs, and emergency nursing.

**Introduction:**

Mass casualty incidents (MCI) are becoming increasingly common and are occurring in locations that have not experienced them previously which adds to the challenge of readiness for emergency departments (EDs). Sporadic occurrences and limited resources add to the complexity of preparing for such an event. In advance of a large gathering in our metropolitan area, we developed and conducted a simulation to better prepare not only our residents, but our MCI planning committee, registered nurses (RNs) and emergency room technicians (ERTs) for an MCI.

Emergency medicine is at the forefront of any hospital’s response to an MCI. These events stretch the resources and force EDs to function differently than usual.^1^ Responding effectively is crucial to minimizing the morbidity and mortality of our patients while maximizing use of available resources. We can improve our level-headedness, efficiency, and department and hospital-level planning through simulation. This has particular implications for residency training with effects on education, preparedness, and wellness.

**Educational Objectives:**

The learners will (1) recognize state of mass casualty exercise as evidenced by verbalization or triaging by START (Simple Triage and Rapid Treatment) criteria, (2) triage several patients, including critically ill or peri-arrest acuities, according to START criteria, (3) recognize the need to limit care based on available resources, as evidenced by verbal orders or communication of priorities to team, and (4) limit emergency resuscitation, given limited resources, by only providing treatments and employing diagnostics that do not deplete limited time, staffing, and space inappropriately.

**Educational Methods:**

A small-scale, high-fidelity simulation was created to replicate the pace and acuity of patients presenting in an MCI. Three critically injured patients with multiple gunshot wounds, represented by high-fidelity manikins with moulaged wounds, were presented over a 6-minute span. The team was allowed 10 minutes total to conduct life-saving measures, targeted evaluation, and disposition of the patients. The simulation was then adapted for use in a second institution’s simulation center to replicate and validate the objectives given a different system.

**Research Methods:**

The learners were immediately verbally debriefed and feedback of the simulation, fidelity and appropriateness of the experience solicited. Unprompted, several of the learners volunteered that the efficacy of the experience was highly educational and valuable. Anonymized digital feedback was requested in the form of an online survey and was generally positive.

The educational content was created by experts in simulation medicine and validated by content experts in the fields of Emergency Medicine, Trauma Surgery and Emergency Nursing.

**Results:**

After the scenario ended, the learners were taken to a second room for debriefing by a trauma surgeon, an emergency medicine attending, and the nurse trauma educator. The actors were able to participate as secondary learners and were rotated out of simulation duties to participate in the debriefing. After this twenty-minute educational debrief, the learners were brought back to the simulation bay and were given a similar scenario. After this iteration, the team debriefed a second time. This hour schedule of cases and debrief was repeated a total of four times with a total of twelve individual learners. Suggestions and verbal feedback were noted for incorporation into appropriate committees or hospital departments. No formal assessment was done and inclusion was strictly on a voluntary basis. An evaluation of the session (on a Likert scale of 1–5) had six respondents which showed an average of 5 on how educational the session was, 4.8 on how realistic the session was, and 4.8 on how effective the session was.

**Discussion:**

Simulation allows participants to safely gain practical experience in MCI management. The experience was well-received, and the learners verbalized increased confidence should they encounter an MCI in the future. We developed this simulation to give residents and nurses first-hand experience performing under high-stress, resource-limited conditions. We also had other learners observing the process which allowed for productive debriefing and planning for improvement. The ideas generated from this ultimately became part of the hospital’s MCI response plan. The main takeaways were triage strategy and limited resource management.

**Topics:**

Mass casualty incident, mass gathering, penetrating trauma, high-fidelity simulation, team-based simulation, trauma center, hospital response planning.

## USER GUIDE

**Table:** 

List of Resources:	
Abstract	1
User Guide	3
Instructor Materials	6
Patient A Instructor Materials	9
Patient A Operator Materials	15
Patient A Simulation Assessment	17
Patient B Instructor Materials	22
Patient B Operator Materials	28
Patient B Simulation Assessment	30
Patient C Instructor Materials	35
Patient C Operator Materials	41
Patient C Simulation Assessment	43
Patient D Instructor Materials	48
Patient D Operator Materials	54
Patient D Simulation Assessment	56
Patient E Instructor Materials	61
Patient E Operator Materials	67
Patient E Simulation Assessment	69
Patient F Instructor Materials	74
Patient F Operator Materials	80
Patient F Simulation Assessment	82
Debriefing and Evaluation Pearls	87
Appendix A: Simulation Events Table, Round 1	90
Appendix B: Simulation Events Table, Round 2	93
Appendix C: Critical Intake – MARCH Algorithm Poster	96
Appendix D: Task Completion Statistics	97
Appendix E: Case Stem	99
Appendix F: Supplies	101
Appendix G: Stimulus Inventor	102
Appendix H: Abbreviation Key	107
Appendix I: Participant Survey for Site 1	108
Appendix J: Participant Survey Results for Site 1	110
Appendix K: Post Simulation Participant Survey Results for Site 2	111

**Learner Audience:**

Interns, Junior Residents, Senior Residents, RNs, Emergency Room Technicians (ERTs), Patient Care Technicians (PCTs)

**Time Required for Implementation:**

Instructor Preparation: 60 minutes

Time for case: 20 minutes of case simulation, 45 minutes total per round

Time for debriefing: 25 minutes, non-consecutive due to logistical constraints of the day and the need to reset the room before round 2 of simulation. Recommend more time if able for final debrief.

**Figure:**
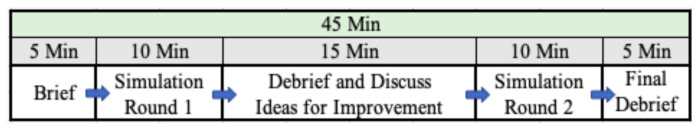


**Recommended Number of Learners per Instructor:**

- 1 resident actively working as team leader per round with 2 rounds per group
- Support team of 2 RNs and 2 ERTs. We kept the same RNs and ERTs throughout all rounds, but they could rotate as well if preferred. These RNs and ERTs can be considered actors to a degree because they were aware of the general flow of cases, but were not fully aware of the injury patterns.
- An alternative team makeup included 1 senior resident MD and 2 trauma RNs. All were considered learners.
- Additional learners can observe but should not assist because the objectives involve working with minimal staffing.

**Topics:**

Mass casualty incident, mass gathering, penetrating trauma, high-fidelity simulation, team-based simulation, trauma center, hospital response planning.

**Objectives:**

By the end of this simulation session, the learner will be able to:

1. Recognize state of mass casualty exercise as evidenced by verbalization or triaging by START criteria.
2. Triage several patients, including critically ill or peri-arrest acuities, according to START criteria.
3. Recognize the need to limit care based on available resources, as evidenced by verbal orders or communication of priorities to team.
4. Limit emergency resuscitation, given limited resources, by only providing treatments and employing diagnostics that do not deplete limited time, staffing, and space inappropriately.

### Linked objectives and methods

Utilizing standardized patients and carrying out large-scale mass casualty drills are expensive, personnel- and resource-intensive, and can interfere with care of actual patients if done *in situ*. Simulation allows participants to efficiently gain practical experience in MCI management while reducing those barriers.

We developed this simulation to give residents and nurses first-hand experience performing under high-stress, resource-limited conditions. Opportunities also exist to allow observation by stakeholders and secondary learners, which augmented debriefings and led to downstream discussions and protocol revisions.

### Recommended pre-reading for instructor

- Menes K, Tintinalli J. How one Las Vegas ED saved hundreds of lives after the worst mass shooting in U.S. history. *Emergency Physicians Monthly*. November 2017. Accessed January 7, 2020. https://epmonthly.com/article/notheroes-wear-capes-one-las-vegas-ed-saved-hundreds-lives-worst-mass-shooting-u-s-history/
- Applicable institution-specific protocols and policies for MCI response in learners’ practice setting.
- Chapter 1 Initial Assessment and Management. In: Merrick C, ed. *Advanced Trauma Life Support: Student Course Manual*, 10th edition. Chicago, Il: American College of Surgeons; 2018: 7–10.
- Margolis A and Millin MG. Chapter 4, “Mass gatherings.” In: Tintinalli JE, Ma OJ, Yealy DM, et al, eds. *Tintinalli’s Emergency Medicine: A Comprehensive Study Guide*. 9^th^ ed. McGraw-Hill Education; 2020. Accessed March 1, 2020.
- Benson M, Koenig KL, Schultz CH. Disaster triage: START, then SAVE-a new method of dynamic triage for victims of a catastrophic earthquake. *Prehospital Disaster Med*. 1996; Apr–Jun; 11(2): 117–24.
- START Adult Triage Algorithm, U.S. Department of Health and Human Services. Chemical Hazards and Emergency Medicine Management. Accessed September 23, 2021. Website. http://chemm.nlm.nih.gov/startadult.htm
- START Triage Flowchart, Critical Illness and Trauma Foundation, Inc. 2001. Accessed March 1, 2021. Website: http://citmt.org/Start/flowchart.htm

### Learner responsible content (optional)

We did not assign any pre-reading for this. However, future sessions may be enhanced by having the residents review their hospital’s MCI plan prior to the session.

### Associated content (optional)

Poster-sized MCI algorithm (See Appendix C).

### Results and tips for successful implementation

This simulation was part of a larger-scale project aimed at developing an emergency department’s response to mass shootings. We found progressively higher-fidelity trial runs to be very helpful prior to the full-scale simulation to identify more obvious issues before involving the learners. We started with a tabletop exercise utilizing a drawing of a patient with multiple penetrating injuries and game pieces to symbolize the team leader, RNs, and ERTs. We walked step-by-step through the process of caring for this hypothetical patient to determine the best ratio of physician to RN to ERT with consideration for the anticipated limitation of available personnel in an MCI. Between performing procedures, placing tourniquets, establishing IVs, and rudimentary documentation, we found the ratio of 1 physician to 2 RNs to 2 ERTs to be most feasible and effective. It is possible that this ratio would vary at other sites based on staffing availability, department layout, etc. At the second site, 1 resident and 2 RNs were a more typical composition for trauma activation. We made a poster outlining a simplified version of the MARCH (Massive hemorrhage, Airway, Respiratory, Circulation, Hypothermia) algorithm, a modified approach to the usual ABCDE (Airway, Breathing, Circulation, Disability, Exposure) format of the primary survey specific to penetrating trauma to increase the team’s efficiency and reduce errors. (See Appendix C).

A trial run of this case for core simulation staff is recommended to optimize feasibility and troubleshoot, given the complicated nature of this simulation. We proceeded with this process using a single patient to verify the feasibility of the process and ensure all required equipment was available. This initial live run resulted in RN-suggested modifications of the one-page MCI documentation form to improve documentation feasibility. The full simulation was then implemented during the emergency medicine residency’s monthly small group conference day. The session took place in the hospital’s dedicated simulation center, facilitated by two dedicated simulationists with several years of experience. Debriefing was done by an EM faculty member (JN). A survey was administered assessing comfort and knowledge of the hospital’s MCI plan before and after the simulation. This survey (on a Likert scale of 1–5) had six respondents, which showed an average of 5 on how educational the session was, 4.8 on how realistic the session was, and 4.8 on how effective the session was (see Appendices I for the survey and J for results).

Each group rotating through our 45-minute station completed two rounds of simulation. For each round, a different second-year EM resident served as team leader. The other residents observed, took notes, and contributed to the debriefing sessions with ideas for improvement and effective approaches that should be encouraged. At the end of the session, residents completed the same questionnaire they had filled out upon arrival.

A variation of this simulation was then done at another EM residency. The second site was a Level 2 Trauma center with a slightly different team makeup that typically included one resident physician and 2 nurses. After consultation with our trauma service, disaster planning committee, and the relevant educators, a replication of simulation was initiated. Medical students and research assistants were recruited to make up for the lack of simulation technicians, and the simulation director acted as the overseer running all three of the monitors and high-fidelity manikins. These medical students or research assistants dressed as EMTs, or wore signs, designated “patient voice.” The patient monitors were configured to be similar to those seen in the trauma bay, replicated on computer screens and run on the Laerdal LLEAP software. A trial run was conducted the night before and the roles of each of the actors reviewed and questions sought and answered. The flow simulation was implemented during the resident and the nurses’ day off. All participants were volunteers who had signed up and had been informed of the location and that it was a trauma scenario. The learners were brought into a room that was arranged to look like a trauma bay as much as possible with three empty gurneys. Patients were brought in according to the schedule and with the same wounds as in the prior simulation. After ten minutes, the scenario was arbitrarily ended and the residents and nurses were taken to a second room for debriefing by a trauma surgeon, an emergency medicine attending, and the nurse trauma educator. The actors were able to participate as secondary learners and would rotate out of simulation duties to participate in the debriefing. After this twenty-minute educational debrief, the learners were brought back to the simulation bay and were given a similar scenario. After this iteration, the team debriefed a second time. This hour schedule of cases and debrief was repeated a total of four times with a total of twelve individual learners. Photographs and videos were taken with the participants’ permission and placed into a video to display their experience as well as showcase the educational endeavor. Feedback was solicited via an anonymous online survey and was generally positive (See Appendix K for full survey results).

Link to our Mass Casualty SIM Training:

https://youtu.be/a16AAAJyxOo

Because this was part of a project to improve the Emergency Department’s MCI plan, the investigators at the first site collected data real-time on the completion of critical actions by each team (Appendix D). Case to case comparisons between sections cannot be made reliably. For instance, Case A was seen as the initial simulation in the first and third sections, but was in the second simulation in the second section. As expected, because of the increasing awareness of the ten-minute time limit, the first of three patients seen by each team had more critical actions completed than the second or third cases seen in each simulation. One exception to this was the last simulation of the day, in which both the first and third patients each had seven critical actions completed. We suspect it is related to cumulative learners and improved efficiency of the nurses and ERTs in the simulation, since this was their sixth iteration of the simulation.

## Supplementary Information











## Figures and Tables

**Table:** 

	Simulation Round 1	Simulation Round 2
Session 1	A B C	D E F
Session 2	D E F	A B C
Session 3	A B C	D E F

**Table:** 

**Vitals:**	Heart Rate (HR) 140	Blood Pressure (BP) 60/palp	Respiratory Rate (RR) 30
	Temperature (T) normal to touch	Oxygen Saturation (O2Sat) undetectable	

**Figure:**
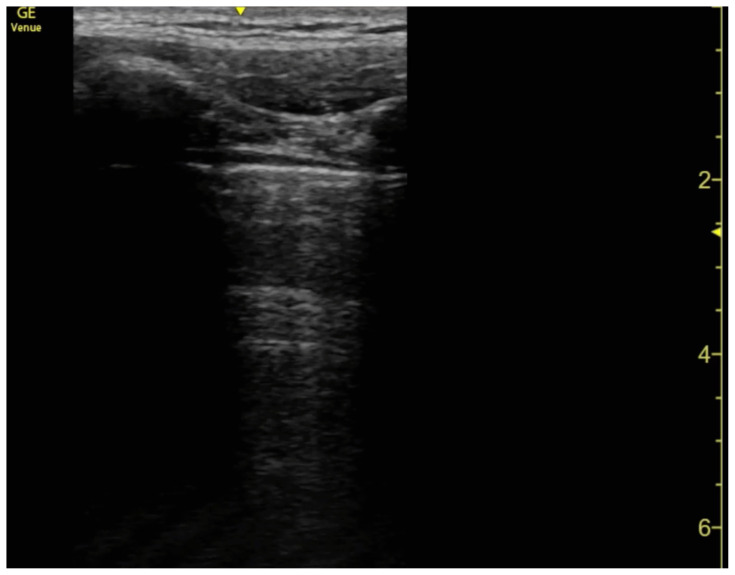


**Figure:**
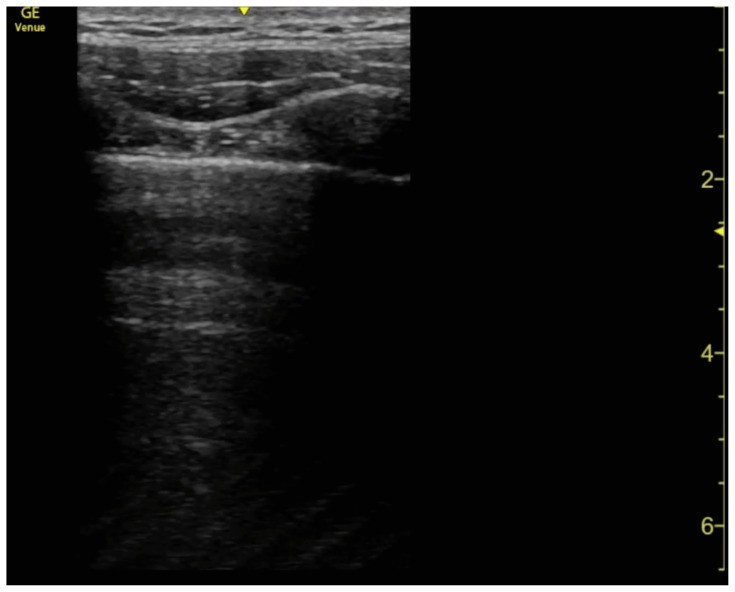


**Figure:**
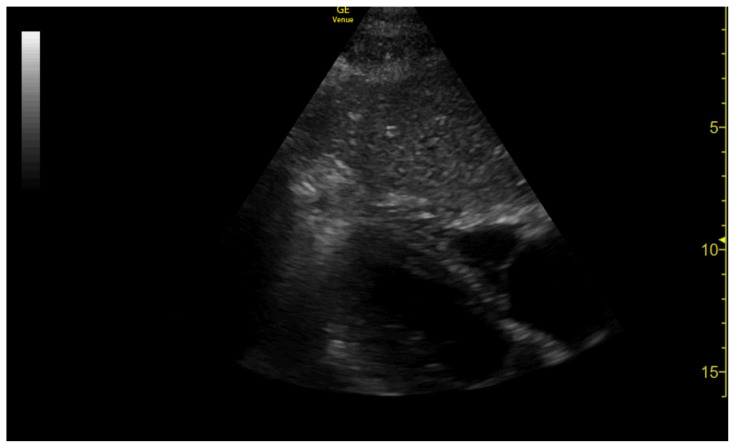


**Figure:**
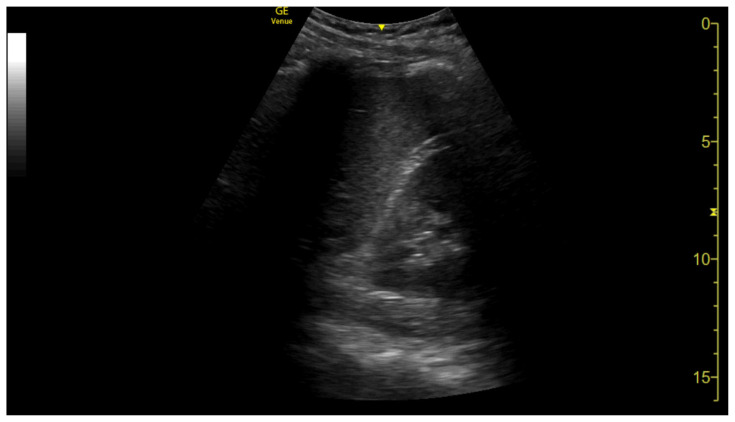


**Table:** SIMULATION EVENTS TABLE:

Minute (state)	Participant action/trigger	Patient status (simulator response) & operator prompts	Monitor display (vital signs)
0:00 (Baseline)	Scenario starts, Participants prepping for multiple patients.	Patient arrives very shortly after scenario starts (within one minute). This patient has a groin wound, a unilateral chest wound, and a head wound. Pt may be unconscious, groan to pain only, flexion of elbows to pain only, eyes remain closed. Not following commands. No recognizable verbalizations.	T normal HR 140 BP 60/palp RR 32 O2 Sat 84% Absent lung sounds on left side with GSW. Absent pulse in leg with groin wound. GCS 6 (E1 V2 M3)
1:00	No pressure on groin wound.	Rapid worsening of vital signs with progression to death (uncontrolled hemorrhage).	HR 170 BP 40/palp RR 30 Rapid progression to PEA (pulseless electrical activity) arrest.
At any point during case.	Intubation with RSI.	Sats to low 90s (if blood pressure is >60/p, otherwise undetectable).	HR 140 BP 70/palp RR bagging O2 Sat 90%
At any point during case.	Decompensating state (Death Spiral): if no interventions initiated or if they release pressure of groin wound (hand/junctional tourniquet or wound packing), every 1-minute drop BP by 10, increase HR by 10, until below is reached).	Drop blood pressure and increase heart rate.	BP 90/60 HR 160 RR 40 O2 Sat 70% GCS: 3 (E1 V1 M1)
At any point during case.	Chest decompression on side of GSW (finger or needle).		Temp 98.0 °F Increase BP to 90/palp Decrease HR to 110 RR 24 O2sat 92% GCS 8 (E2 V2 M4)
At any point during case.	Arrest state.	If the patient has been at the lowest point of the “death spiral” for 1 minute, he goes into a PEA arrest until 2 units of blood are transfused and pressure maintained on the groin wound (unlikely to happen in this case).	
At any point during case.	Transfuse blood or give crystalloid.	BP and HR further improve.	
(Case Completion)	Physician decides on disposition.	Move the patient out of the room. Assume any disposition option is ready for the patient.	

**Table:** 

**Critical Actions:** Place direct pressure over groin wound Establish IV or IO access Give RSI (rapid sequence induction) meds to facilitate intubation Intubate or place supraglottic airway Decompress chest on the left side of the GSW Transfuse blood products per local protocol Give TXA (Tranexamic acid) Place pressure dressing over groin wound Decide disposition for patient	0:00

**Table:** 

	Milestone	Did not achieve level 1	Level 1	Level 2	Level 3
1	**Emergency Stabilization (PC1)**	□ Did not achieve Level 1	□ Recognizes abnormal vital signs	□ Recognizes an unstable patient, requiring intervention Performs primary assessment Discerns data to formulate a diagnostic impression/plan	□ Manages and prioritizes critical actions in a critically ill patient Reassesses after implementing a stabilizing intervention
2	**Performance of focused history and physical (PC2)**	□ Did not achieve Level 1	□ Performs a reliable, comprehensive history and physical exam	□ Performs and communicates a focused history and physical exam based on chief complaint and urgent issues	□ Prioritizes essential components of history and physical exam given dynamic circumstances
3	**Diagnostic studies (PC3)**	□ Did not achieve Level 1	□ Determines the necessity of diagnostic studies	□ Orders appropriate diagnostic studies. Performs appropriate bedside diagnostic studies/procedures	□ Prioritizes essential testing Interprets results of diagnostic studies Reviews risks, benefits, contraindications, and alternatives to a diagnostic study or procedure
4	**Diagnosis (PC4)**	□ Did not achieve Level 1	□ Considers a list of potential diagnoses	□ Considers an appropriate list of potential diagnosis May or may not make correct diagnosis	□ Makes the appropriate diagnosis Considers other potential diagnoses, avoiding premature closure
5	**Pharmacotherapy (PC5)**	□ Did not achieve Level 1	□ Asks patient for drug allergies	□ Selects an medication for therapeutic intervention, consider potential adverse effects	□ Selects the most appropriate medication and understands mechanism of action, effect, and potential side effects Considers and recognizes drug-drug interactions
6	**Observation and reassessment (PC6)**	□ Did not achieve Level 1	□ Reevaluates patient at least one time during case	□ Reevaluates patient after most therapeutic interventions	□ Consistently evaluates the effectiveness of therapies at appropriate intervals
7	**Disposition (PC7)**	□ Did not achieve Level 1	□ Appropriately selects whether to admit or discharge the patient	□ Appropriately selects whether to admit or discharge Involves the expertise of some of the appropriate specialists	□ Educates the patient appropriately about their disposition Assigns patient to an appropriate level of care (ICU/Tele/Floor) Involves expertise of all appropriate specialists
9	**General Approach to Procedures (PC9)**	□ Did not achieve Level 1	□ Identifies pertinent anatomy and physiology for a procedure Uses appropriate Universal Precautions	□ Obtains informed consent Knows indications, contraindications, anatomic landmarks, equipment, anesthetic and procedural technique, and potential complications for common ED procedures	□ Determines a back-up strategy if initial attempts are unsuccessful Correctly interprets results of diagnostic procedure
20	**Professional Values (PROF1)**	□ Did not achieve Level 1	□ Demonstrates caring, honest behavior	□ Exhibits compassion, respect, sensitivity and responsiveness	□ Develops alternative care plans when patients’ personal beliefs and decisions preclude standard care
22	**Patient centered communication (ICS1)**	□ Did not achieve level 1	□ Establishes rapport and demonstrates empathy to patient (and family) Listens effectively	□ Elicits patient’s reason for seeking health care	□ Manages patient expectations in a manner that minimizes potential for stress, conflict, and misunderstanding. Effectively communicates with vulnerable populations, (at risk patients and families)
23	**Team management (ICS2)**	□ Did not achieve level 1	□ Recognizes other members of the patient care team during case (nurse, techs)	□ Communicates pertinent information to other healthcare colleagues	□ Communicates a clear, succinct, and appropriate handoff with specialists and other colleagues Communicates effectively with ancillary staff

**Table:** 

**Vitals:**	Heart Rate (HR) 130	Blood Pressure (BP) 90/palp	Respiratory Rate (RR) 28
	Temperature (T) normal to touch	Oxygen Saturation (O2Sat) 88%	

**Figure:**
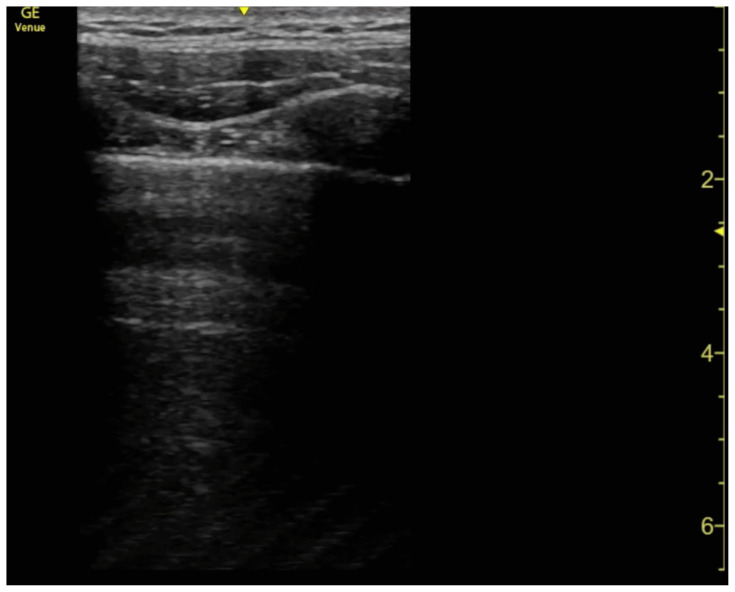


**Figure:**
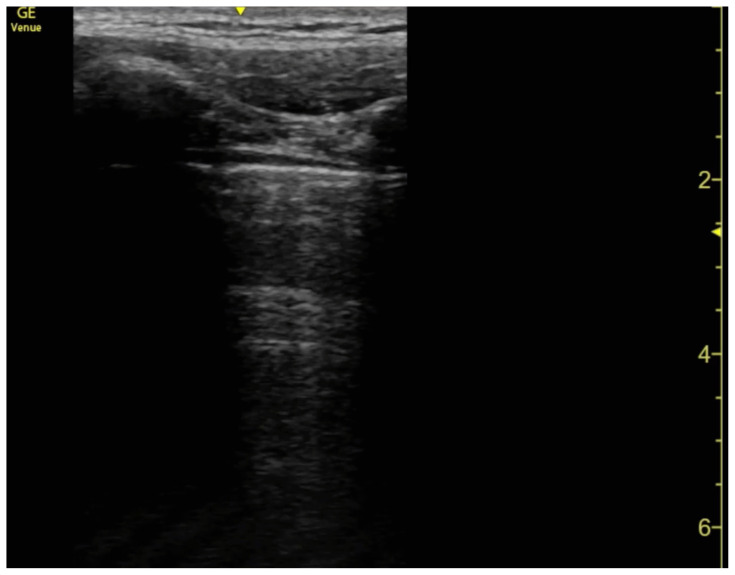


**Figure:**
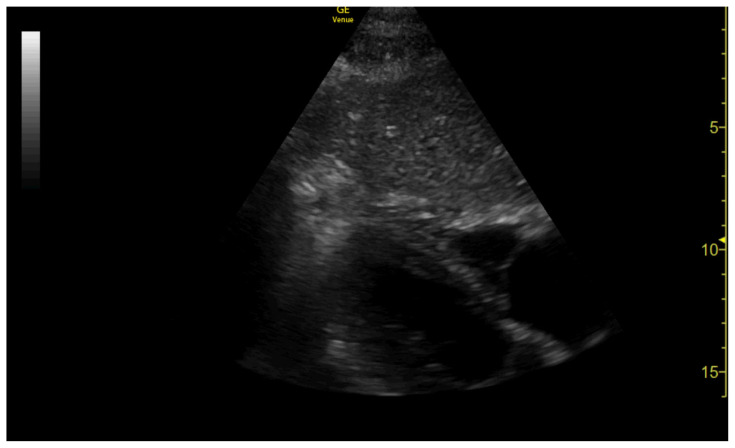


**Figure:**
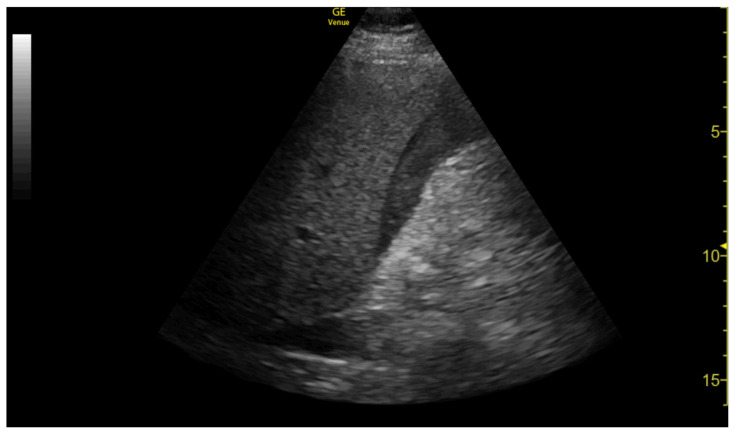


**Table:** SIMULATION EVENTS TABLE:

Minute (state)	Participant action/trigger	Patient status (simulator response) & operator prompts	Monitor display (vital signs)
3:00 (Baseline)		Three min after scenario start, this patient arrives regardless of team readiness. This patient has one GSW to a thoracoabdominal region and one to a lower leg with catastrophic injury or amputation. Pt is awake and voice confused and screaming, “I can’t …breathe!” “My…leg! My…baby.” “Where’s….my…baby?”	T normal Temp 97.0°F HR 130 BP 90/palp RR 28 O2 Sat 88% on facemask. Pulsatile bleeding from lower leg. Decreased lung sounds on side of GSW. GCS 12 (E4 V4 M4)
	Participants tighten the lower extremity tourniquet.	Bleeding in leg stops. Vitals improve.	HR 120 BP 80/palp
	Chest decompression state (after tube is placed).	Breathing improves, BP increases. After a minute, patient will start reaching toward chest tube to pull it out--move the manikin’s hand in that direction and say, “the patient seems to be grabbing toward the chest tube.” If they do not respond within 2 minutes, pull the chest tube out and start “death spiral.”	Temp 98 °F BP 90/palp HR 100 RRL 24 O2 Sat 95% GCS 13 (E4 V4 M5)
	Transfuse blood or give crystalloid.	BP and HR further improve.	
	POCUS exam.	Abnormal lung sliding on side of injury, positive abdominal free fluid on side of injury.	
	Arrest State.	If the patient has been at the lowest point of the “death spiral” for 1 minute, goes into a PEA arrest until 2 units of blood are transfused, tourniquet placed and chest tubes inserted (unlikely to happen in this case).	
	Decompensating state (“death spiral”):	If no interventions initiated or if they do not put a tourniquet on wound, every 1minute, drop BP by 10, increase, HR by 10, etc. until below is reached).	Temp 95.0 °F BP 40/palp, HR 160 RR 40 O2 Sat 70% GCS 3 (E1 V1 M1)
(Case Completion)	Physician decides on disposition or 10 minutes from scenario start.	Move patient out of room. Assume any disposition option is ready for the patient.	

**Table:** 

**Critical Actions:** Adjust tourniquet on leg to stop blood flow at site of amputation Establish IV or IO access Give RSI meds to facilitate intubation Intubate or place supraglottic airway Decompress chest on the right side of the GSW Perform point-of-care ultrasound (POCUS) examination Transfuse blood products per local protocol Give TXA Decide disposition for patient	0:00

**Table:** 

	Milestone	Did not achieve level 1	Level 1	Level 2	Level 3
1	**Emergency Stabilization (PC1)**	□ Did not achieve Level 1	□ Recognizes abnormal vital signs	□ Recognizes an unstable patient, requiring intervention Performs primary assessment Discerns data to formulate a diagnostic impression/plan	□ Manages and prioritizes critical actions in a critically ill patient Reassesses after implementing a stabilizing intervention
2	**Performance of focused history and physical (PC2)**	□ Did not achieve Level 1	□ Performs a reliable, comprehensive history and physical exam	□ Performs and communicates a focused history and physical exam based on chief complaint and urgent issues	□ Prioritizes essential components of history and physical exam given dynamic circumstances
3	**Diagnostic studies (PC3)**	□ Did not achieve Level 1	□ Determines the necessity of diagnostic studies	□ Orders appropriate diagnostic studies. Performs appropriate bedside diagnostic studies/procedures	□ Prioritizes essential testing Interprets results of diagnostic studies Reviews risks, benefits, contraindications, and alternatives to a diagnostic study or procedure
4	**Diagnosis (PC4)**	□ Did not achieve Level 1	□ Considers a list of potential diagnoses	□ Considers an appropriate list of potential diagnosis May or may not make correct diagnosis	□ Makes the appropriate diagnosis Considers other potential diagnoses, avoiding premature closure
5	**Pharmacotherapy (PC5)**	□ Did not achieve Level 1	□ Asks patient for drug allergies	□ Selects an medication for therapeutic intervention, consider potential adverse effects	□ Selects the most appropriate medication and understands mechanism of action, effect, and potential side effects Considers and recognizes drug-drug interactions
6	**Observation and reassessment (PC6)**	□ Did not achieve Level 1	□ Reevaluates patient at least one time during case	□ Reevaluates patient after most therapeutic interventions	□ Consistently evaluates the effectiveness of therapies at appropriate intervals
7	**Disposition (PC7)**	□ Did not achieve Level 1	□ Appropriately selects whether to admit or discharge the patient	□ Appropriately selects whether to admit or discharge Involves the expertise of some of the appropriate specialists	□ Educates the patient appropriately about their disposition Assigns patient to an appropriate level of care (ICU/Tele/Floor) Involves expertise of all appropriate specialists
9	**General Approach to Procedures (PC9)**	□ Did not achieve Level 1	□ Identifies pertinent anatomy and physiology for a procedure Uses appropriate Universal Precautions	□ Obtains informed consent Knows indications, contraindications, anatomic landmarks, equipment, anesthetic and procedural technique, and potential complications for common ED procedures	□ Determines a back-up strategy if initial attempts are unsuccessful Correctly interprets results of diagnostic procedure
20	**Professional Values (PROF1)**	□ Did not achieve Level 1	□ Demonstrates caring, honest behavior	□ Exhibits compassion, respect, sensitivity and responsiveness	□ Develops alternative care plans when patients’ personal beliefs and decisions preclude standard care
22	**Patient centered communication (ICS1)**	□ Did not achieve level 1	□ Establishes rapport and demonstrates empathy to patient (and family) Listens effectively	□ Elicits patient’s reason for seeking health care	□ Manages patient expectations in a manner that minimizes potential for stress, conflict, and misunderstanding. Effectively communicates with vulnerable populations, (at risk patients and families)
23	**Team management (ICS2)**	□ Did not achieve level 1	□ Recognizes other members of the patient care team during case (nurse, techs)	□ Communicates pertinent information to other healthcare colleagues	□ Communicates a clear, succinct, and appropriate handoff with specialists and other colleagues Communicates effectively with ancillary staff

**Table:** 

**Vitals:**	Heart Rate (HR) 50	Blood Pressure (BP) 40/palp	Temperature (T) normal to touch
	Oxygen Saturation (O2Sat) undetectable		

**Figure:**
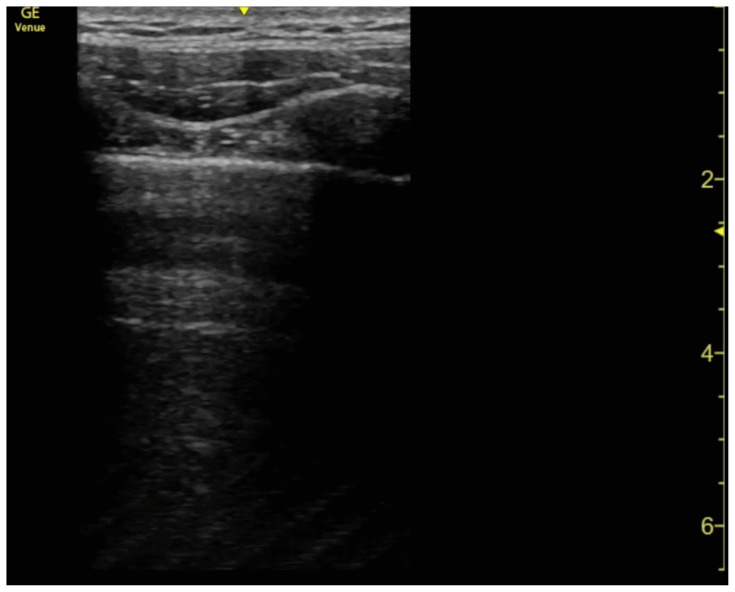


**Figure:**
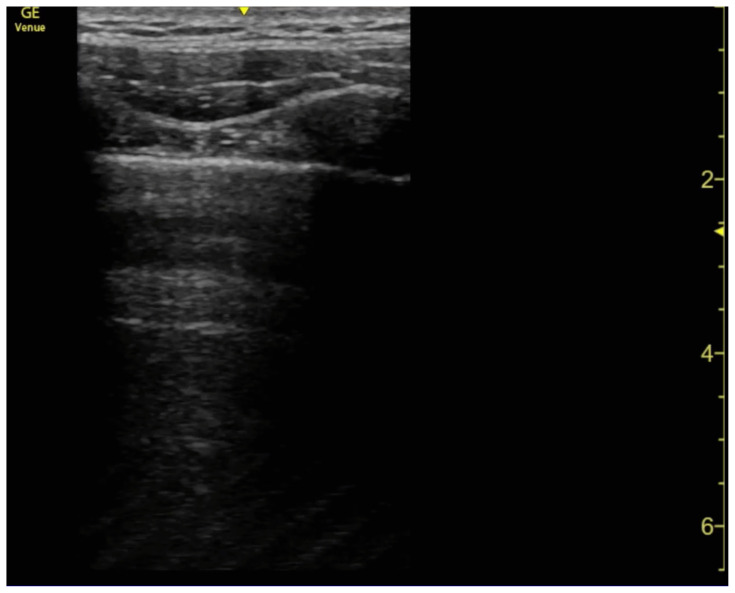


**Figure:**
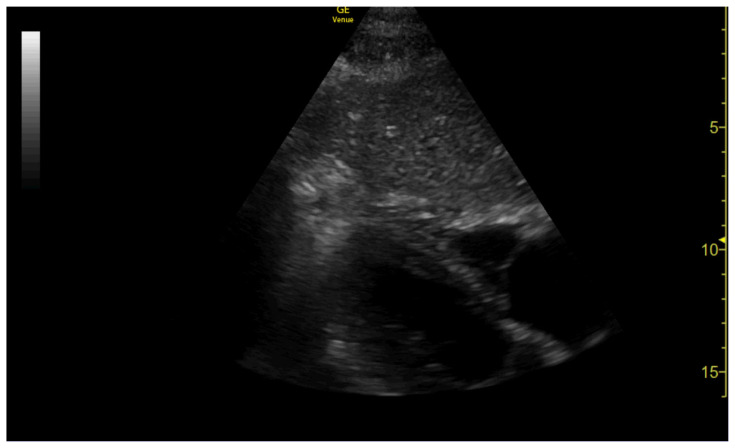


**Figure:**
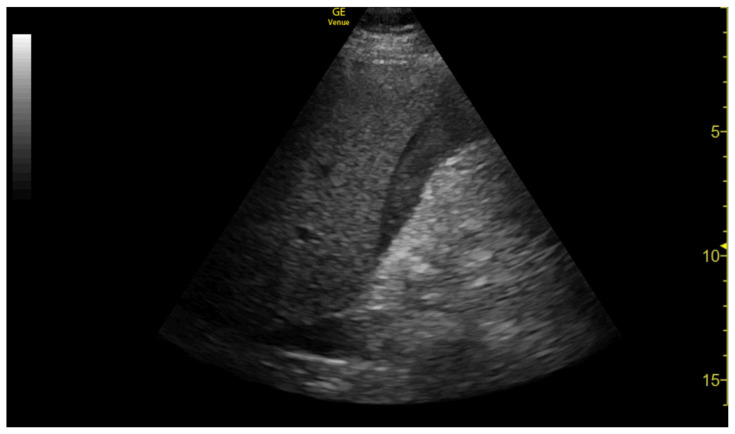


**Table:** SIMULATION EVENTS TABLE:

Minute (state)	Participant action/trigger	Patient status (simulator response) & operator prompts	Monitor display (vital signs)
6:00 (Baseline)		Six min after scenario start, this patient arrives regardless of team readiness. This patient is a child with a GSW to one side of the neck and one GSW to the abdomen. Unconscious, unresponsive, eyes remain closed. Copious amounts of blood around the neck. No verbalizations.	T cool to touch Temp 96.0 °F HR 40 (thready central pulse) BP undetectable RR agonal O2 Sat undetectable GCS 3 (E1 V1 M1)
	Pressure on neck wound.	Bleeding stops, but no improvement in vital signs.	
7:30		Patient develops PEA arrest. After 1–2 minutes, the patient loses pulses and stops breathing. No amount of resuscitation will revive the patient.	BP 0
	Intubation and/or Chest compressions.	No change in vitals.	
		Pt will continue to worsen and die, regardless of the team’s interventions.	
(Case Completion)	Physician stops resuscitative efforts or 10 minutes from scenario start.		

**Table:** 

**Critical Actions:** Place direct pressure on neck wound Establish IV or IO access Perform POCUS exam Intubate or place supraglottic airway Stop resuscitative efforts and transfer to areas for expectant care or deceased patients	0:00

**Table:** 

	Milestone	Did not achieve level 1	Level 1	Level 2	Level 3
1	**Emergency Stabilization (PC1)**	□ Did not achieve Level 1	□ Recognizes abnormal vital signs	□ Recognizes an unstable patient, requiring intervention Performs primary assessment Discerns data to formulate a diagnostic impression/plan	□ Manages and prioritizes critical actions in a critically ill patient Reassesses after implementing a stabilizing intervention
2	**Performance of focused history and physical (PC2)**	□ Did not achieve Level 1	□ Performs a reliable, comprehensive history and physical exam	□ Performs and communicates a focused history and physical exam based on chief complaint and urgent issues	□ Prioritizes essential components of history and physical exam given dynamic circumstances
3	**Diagnostic studies (PC3)**	□ Did not achieve Level 1	□ Determines the necessity of diagnostic studies	□ Orders appropriate diagnostic studies. Performs appropriate bedside diagnostic studies/procedures	□ Prioritizes essential testing Interprets results of diagnostic studies Reviews risks, benefits, contraindications, and alternatives to a diagnostic study or procedure
4	**Diagnosis (PC4)**	□ Did not achieve Level 1	□ Considers a list of potential diagnoses	□ Considers an appropriate list of potential diagnosis May or may not make correct diagnosis	□ Makes the appropriate diagnosis Considers other potential diagnoses, avoiding premature closure
5	**Pharmacotherapy (PC5)**	□ Did not achieve Level 1	□ Asks patient for drug allergies	□ Selects an medication for therapeutic intervention, consider potential adverse effects	□ Selects the most appropriate medication and understands mechanism of action, effect, and potential side effects Considers and recognizes drug-drug interactions
6	**Observation and reassessment (PC6)**	□ Did not achieve Level 1	□ Reevaluates patient at least one time during case	□ Reevaluates patient after most therapeutic interventions	□ Consistently evaluates the effectiveness of therapies at appropriate intervals
7	**Disposition (PC7)**	□ Did not achieve Level 1	□ Appropriately selects whether to admit or discharge the patient	□ Appropriately selects whether to admit or discharge Involves the expertise of some of the appropriate specialists	□ Educates the patient appropriately about their disposition Assigns patient to an appropriate level of care (ICU/Tele/Floor) Involves expertise of all appropriate specialists
9	**General Approach to Procedures (PC9)**	□ Did not achieve Level 1	□ Identifies pertinent anatomy and physiology for a procedure Uses appropriate Universal Precautions	□ Obtains informed consent Knows indications, contraindications, anatomic landmarks, equipment, anesthetic and procedural technique, and potential complications for common ED procedures	□ Determines a back-up strategy if initial attempts are unsuccessful Correctly interprets results of diagnostic procedure
20	**Professional Values (PROF1)**	□ Did not achieve Level 1	□ Demonstrates caring, honest behavior	□ Exhibits compassion, respect, sensitivity and responsiveness	□ Develops alternative care plans when patients’ personal beliefs and decisions preclude standard care
22	**Patient centered communication (ICS1)**	□ Did not achieve level 1	□ Establishes rapport and demonstrates empathy to patient (and family) Listens effectively	□ Elicits patient’s reason for seeking health care	□ Manages patient expectations in a manner that minimizes potential for stress, conflict, and misunderstanding. Effectively communicates with vulnerable populations, (at risk patients and families)
23	**Team management (ICS2)**	□ Did not achieve level 1	□ Recognizes other members of the patient care team during case (nurse, techs)	□ Communicates pertinent information to other healthcare colleagues	□ Communicates a clear, succinct, and appropriate handoff with specialists and other colleagues Communicates effectively with ancillary staff

**Table:** 

**Vitals:**	Heart Rate (HR) 140	Blood Pressure (BP) 60/palp	Respiratory Rate (RR) 30
	Temperature (T) normal to touch	Oxygen Saturation (O2Sat) undetectable	

**Figure:**
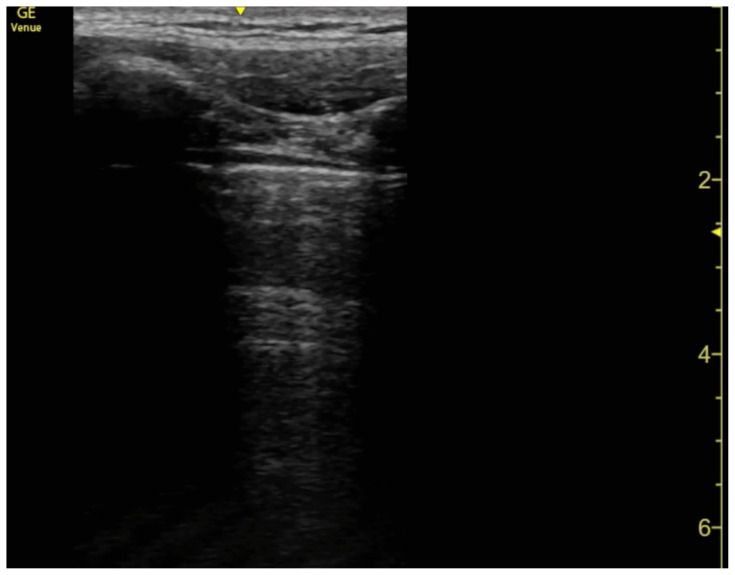


**Figure:**
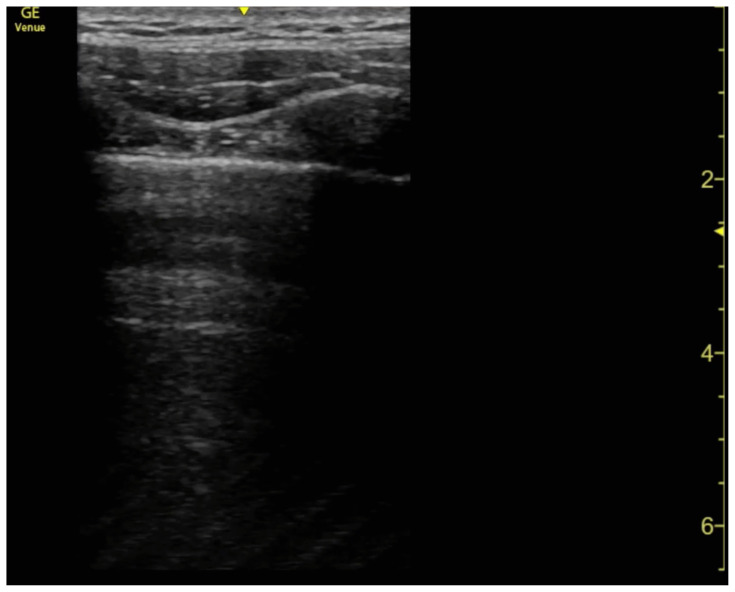


**Figure:**
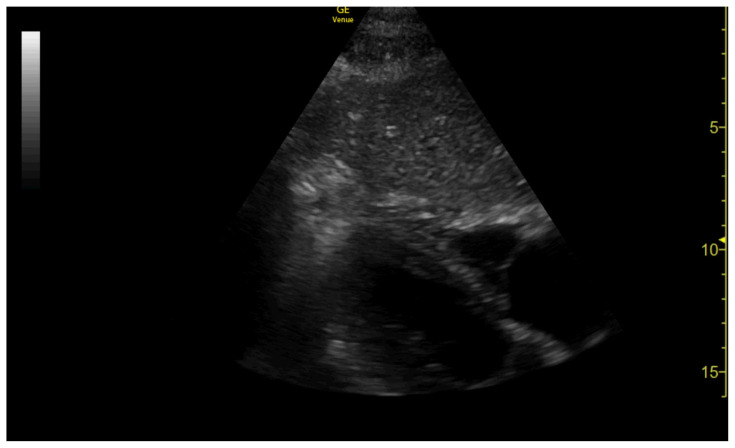


**Figure:**
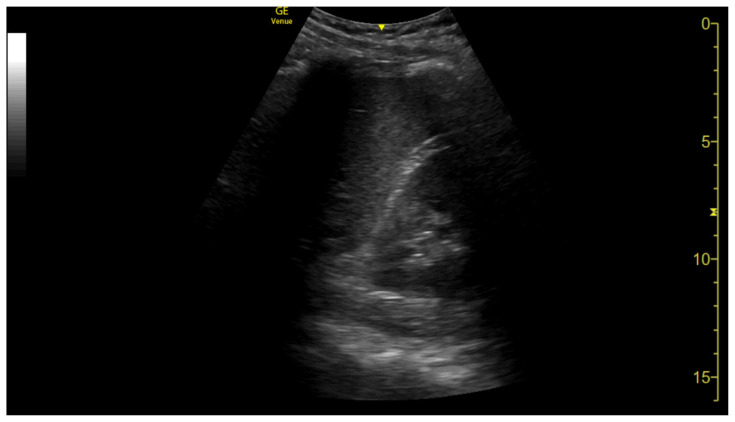


**Table:** SIMULATION EVENTS TABLE:

Minute (state)	Participant action/trigger	Patient status (simulator response) & operator prompts	Monitor display (vital signs)
0:00 (Baseline)	Scenario starts. Participants prepping for multiple patients.	Patient arrives very shortly after scenario starts (within one minute). This patient has a neck wound and a unilateral chest wound. Pt is unconscious, groaning. Flexion of elbows to pain only, eyes remain closed. Barely awake. No recognizable verbalizations.	T normal HR 140 BP 60/palp RR 32 O2 Sat undetectable Absent lung sounds on side of chest wound, GCS 6 (E1 V2 M3)
1:00	Decompensating state (death spiral).	If no interventions initiated or if they let up pressure on neck wound, every 1 minute drop BP by 10, increase HR by 10, etc. until below is reached). Start rapid progression to death (bleeding out).	Temp 95.0 °F BP 40/palp HR 160 RR 40 O2 Sat 70% GCS 3 (E1 V1 M1)
At any point during case.	Release pressure on neck wound (hand or wound packing).	Drop blood pressure and increase heart rate.	HR 170 BP 40/palp RR 32 O2 Sat undetectable
At any point during case.	Transfuse blood or give crystalloid.	BP and HR further improve.	HR 120 BP 70/palp RR 32 O2 Sat undetectable
At any point during case.	Intubation with RSI or surgical airway.	Sats to low 90s (if blood pressure is >60/p, otherwise undetectable).	HR 120 BP 70/palp RR bagging O2 Sat 91%
At any point during case.	Chest decompression on side of GSW (finger or needle).	Increase blood pressure to 90/p and decrease HR to 110.	Temp 98.0 °F BP 90/60 HR 110 RR 24 O2 Sat 92% GCS 8 (E2 V2 M4)
	Arrest state.	If the patient has been at the lowest point of the death spiral for 1 minute, he goes into a PEA arrest until 2 units of blood are transfused and pressure maintained on neck wound.	
(Case Completion)	Physician decides on disposition.	Move patient out of room. Assume any disposition option is ready for the patient.	

**Table:** 

**Critical Actions:** Place direct pressure over neck wound Establish IV or IO access Give RSI meds to facilitate intubation Intubate or place supraglottic airway Decompress chest on the side of the GSW Transfuse blood products per local protocol Give TXA Place pressure dressing over neck wound Decide disposition for patient	0:00

**Table:** 

	Milestone	Did not achieve level 1	Level 1	Level 2	Level 3
1	**Emergency Stabilization (PC1)**	□ Did not achieve Level 1	□ Recognizes abnormal vital signs	□ Recognizes an unstable patient, requiring intervention Performs primary assessment Discerns data to formulate a diagnostic impression/plan	□ Manages and prioritizes critical actions in a critically ill patient Reassesses after implementing a stabilizing intervention
2	**Performance of focused history and physical (PC2)**	□ Did not achieve Level 1	□ Performs a reliable, comprehensive history and physical exam	□ Performs and communicates a focused history and physical exam based on chief complaint and urgent issues	□ Prioritizes essential components of history and physical exam given dynamic circumstances
3	**Diagnostic studies (PC3)**	□ Did not achieve Level 1	□ Determines the necessity of diagnostic studies	□ Orders appropriate diagnostic studies. Performs appropriate bedside diagnostic studies/procedures	□ Prioritizes essential testing Interprets results of diagnostic studies Reviews risks, benefits, contraindications, and alternatives to a diagnostic study or procedure
4	**Diagnosis (PC4)**	□ Did not achieve Level 1	□ Considers a list of potential diagnoses	□ Considers an appropriate list of potential diagnosis May or may not make correct diagnosis	□ Makes the appropriate diagnosis Considers other potential diagnoses, avoiding premature closure
5	**Pharmacotherapy (PC5)**	□ Did not achieve Level 1	□ Asks patient for drug allergies	□ Selects an medication for therapeutic intervention, consider potential adverse effects	□ Selects the most appropriate medication and understands mechanism of action, effect, and potential side effects Considers and recognizes drug-drug interactions
6	**Observation and reassessment (PC6)**	Did not achieve Level 1	□ Reevaluates patient at least one time during case	□ Reevaluates patient after most therapeutic interventions	□ Consistently evaluates the effectiveness of therapies at appropriate intervals
7	**Disposition (PC7)**	□ Did not achieve Level 1	□ Appropriately selects whether to admit or discharge the patient	□ Appropriately selects whether to admit or discharge Involves the expertise of some of the appropriate specialists	□ Educates the patient appropriately about their disposition Assigns patient to an appropriate level of care (ICU/Tele/Floor) Involves expertise of all appropriate specialists
9	**General Approach to Procedures (PC9)**	□ Did not achieve Level 1	□ Identifies pertinent anatomy and physiology for a procedure Uses appropriate Universal Precautions	□ Obtains informed consent Knows indications, contraindications, anatomic landmarks, equipment, anesthetic and procedural technique, and potential complications for common ED procedures	□ Determines a back-up strategy if initial attempts are unsuccessful Correctly interprets results of diagnostic procedure
20	**Professional Values (PROF1)**	□ Did not achieve Level 1	□ Demonstrates caring, honest behavior	□ Exhibits compassion, respect, sensitivity and responsiveness	□ Develops alternative care plans when patients’ personal beliefs and decisions preclude standard care
22	**Patient centered communication (ICS1)**	□ Did not achieve level 1	□ Establishes rapport and demonstrates empathy to patient (and family) Listens effectively	□ Elicits patient’s reason for seeking health care	□ Manages patient expectations in a manner that minimizes potential for stress, conflict, and misunderstanding. Effectively communicates with vulnerable populations, (at risk patients and families)
23	**Team management (ICS2)**	□ Did not achieve level 1	□ Recognizes other members of the patient care team during case (nurse, techs)	□ Communicates pertinent information to other healthcare colleagues	□ Communicates a clear, succinct, and appropriate handoff with specialists and other colleagues Communicates effectively with ancillary staff

**Table:** 

**Vitals:**	Heart Rate (HR) 140	Blood Pressure (BP) 70/palp	Respiratory Rate (RR) 28
	Temperature (T) normal to touch	Oxygen Saturation (O_2_Sat) undetectable	

**Figure:**
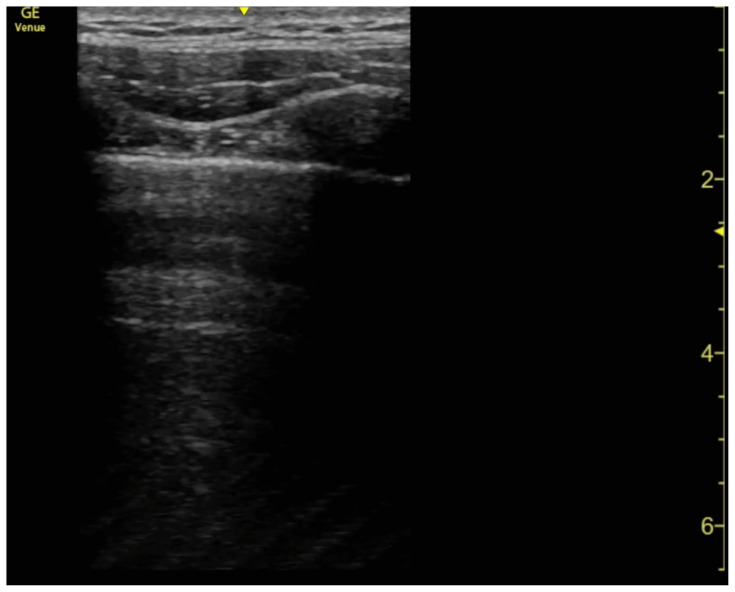


**Figure:**
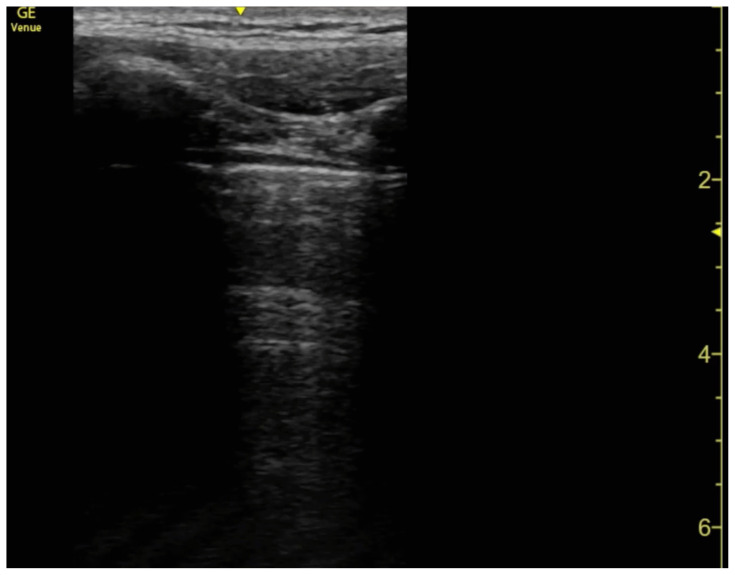


**Figure:**
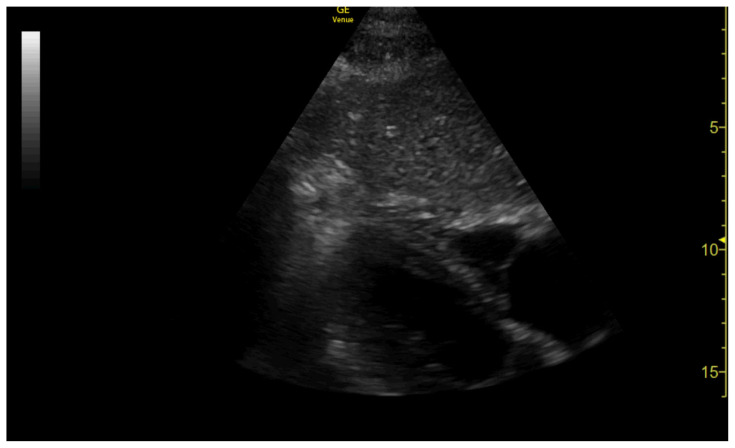


**Figure:**
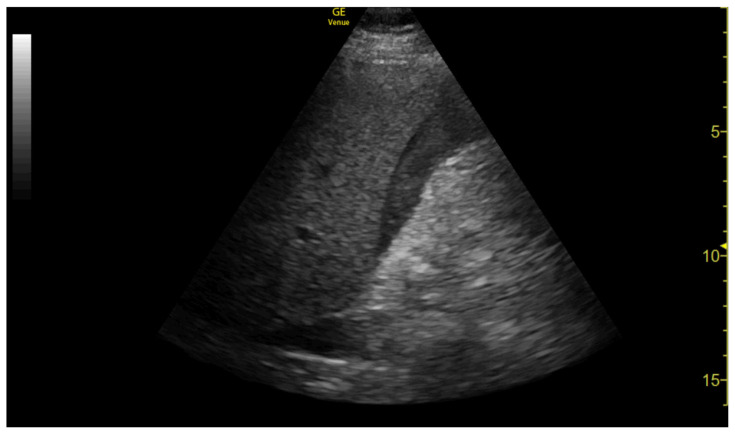


**Table:** SIMULATION EVENTS TABLE:

Minute (state)	Participant action/trigger	Patient status (simulator response) & operator prompts	Monitor display (vital signs)
3:00 (Baseline)		Three min after scenario start, this patient arrives regardless of team readiness. This patient has one GSW to the head and another to a unilateral thoracoabdominal region. Unconscious, groans to pain only, flexion of elbows to pain only, eyes remain closed.	Temp 97.0 °F HR 140 BP 70/palp RR 40 O2 Sat 80% GCS 6 (E1 V2 M3)
	Chest decompression.	Breathing improves, BP increases, Decrease HR.	HR 120 BP 85/palp O2 Sat 90% RR 24 GCS 8 (E2 V2 M4)
	Transfuse blood or give crystalloid.	BP and HR further improve.	HR 110 BP 90/40
	POCUS exam.	Abnormal lung sliding on side of injury, positive abdominal free fluid on side of injury.	
	Intubation via RSI.	Sats further improve.	O2 sats 95%
	Decompensating state (“death spiral”).	If no interventions initiated, every 1 minute drop BP by 10, increase HR by 10, etc. until below is reached.	Temp 95 °F BP 40/palp HR 110 RR 40 O2 Sat 70% GCS 3 (E1 V1 M1)
	Arrest state.	If the patient has been at the lowest point of the death spiral for 1 minute, he goes into a PEA arrest until 2 units of blood are transfused and chest tube placed.	
(Case Completion)	Physician decides on disposition or 10 minutes from start of case.	Move the patient out of the room. Assume any disposition option is ready for the patient.	

**Table:** 

**Critical Actions:** Establish IV or IO access Give RSI meds to facilitate intubation Intubate or place supraglottic airway Decompress chest on the right side of the GSW Transfuse blood products per local protocol Give TXA Decide disposition for patient	0:00

**Table:** 

	Milestone	Did not achieve level 1	Level 1	Level 2	Level 3
1	**Emergency Stabilization (PC1)**	□ Did not achieve Level 1	□ Recognizes abnormal vital signs	□ Recognizes an unstable patient, requiring intervention Performs primary assessment Discerns data to formulate a diagnostic impression/plan	□ Manages and prioritizes critical actions in a critically ill patient Reassesses after implementing a stabilizing intervention
2	**Performance of focused history and physical (PC2)**	□ Did not achieve Level 1	□ Performs a reliable, comprehensive history and physical exam	□ Performs and communicates a focused history and physical exam based on chief complaint and urgent issues	□ Prioritizes essential components of history and physical exam given dynamic circumstances
3	**Diagnostic studies (PC3)**	□ Did not achieve Level 1	□ Determines the necessity of diagnostic studies	□ Orders appropriate diagnostic studies. Performs appropriate bedside diagnostic studies/procedures	□ Prioritizes essential testing Interprets results of diagnostic studies Reviews risks, benefits, contraindications, and alternatives to a diagnostic study or procedure
4	**Diagnosis (PC4)**	□ Did not achieve Level 1	□ Considers a list of potential diagnoses	□ Considers an appropriate list of potential diagnosis May or may not make correct diagnosis	□ Makes the appropriate diagnosis Considers other potential diagnoses, avoiding premature closure
5	**Pharmacotherapy (PC5)**	□ Did not achieve Level 1	□ Asks patient for drug allergies	□ Selects an medication for therapeutic intervention, consider potential adverse effects	□ Selects the most appropriate medication and understands mechanism of action, effect, and potential side effects Considers and recognizes drug-drug interactions
6	**Observation and reassessment (PC6)**	□ Did not achieve Level 1	□ Reevaluates patient at least one time during case	□ Reevaluates patient after most therapeutic interventions	□ Consistently evaluates the effectiveness of therapies at appropriate intervals
7	**Disposition (PC7)**	□ Did not achieve Level 1	□ Appropriately selects whether to admit or discharge the patient	□ Appropriately selects whether to admit or discharge Involves the expertise of some of the appropriate specialists	Educates the patient appropriately about their disposition Assigns patient to an appropriate level of care (ICU/Tele/Floor) Involves expertise of all appropriate specialists
9	**General Approach to Procedures (PC9)**	□ Did not achieve Level 1	□ Identifies pertinent anatomy and physiology for a procedure Uses appropriate Universal Precautions	□ Obtains informed consent Knows indications, contraindications, anatomic landmarks, equipment, anesthetic and procedural technique, and potential complications for common ED procedures	□ Determines a back-up strategy if initial attempts are unsuccessful Correctly interprets results of diagnostic procedure
20	**Professional Values (PROF1)**	□ Did not achieve Level 1	□ Demonstrates caring, honest behavior	□ Exhibits compassion, respect, sensitivity and responsiveness	□ Develops alternative care plans when patients’ personal beliefs and decisions preclude standard care
22	**Patient centered communication (ICS1)**	□ Did not achieve level 1	□ Establishes rapport and demonstrates empathy to patient (and family) Listens effectively	□ Elicits patient’s reason for seeking health care	□ Manages patient expectations in a manner that minimizes potential for stress, conflict, and misunderstanding. Effectively communicates with vulnerable populations, (at risk patients and families)
23	**Team management (ICS2)**	□ Did not achieve level 1	□ Recognizes other members of the patient care team during case (nurse, techs)	□ Communicates pertinent information to other healthcare colleagues	□ Communicates a clear, succinct, and appropriate handoff with specialists and other colleagues Communicates effectively with ancillary staff

**Table:** 

**Vitals:**	Heart Rate (HR) 140	Blood Pressure (BP) 60/palp	Respiratory Rate (RR) 35
	Temperature (T) normal to touch	Oxygen Saturation (O_2_Sat) undetectable	

**Figure:**
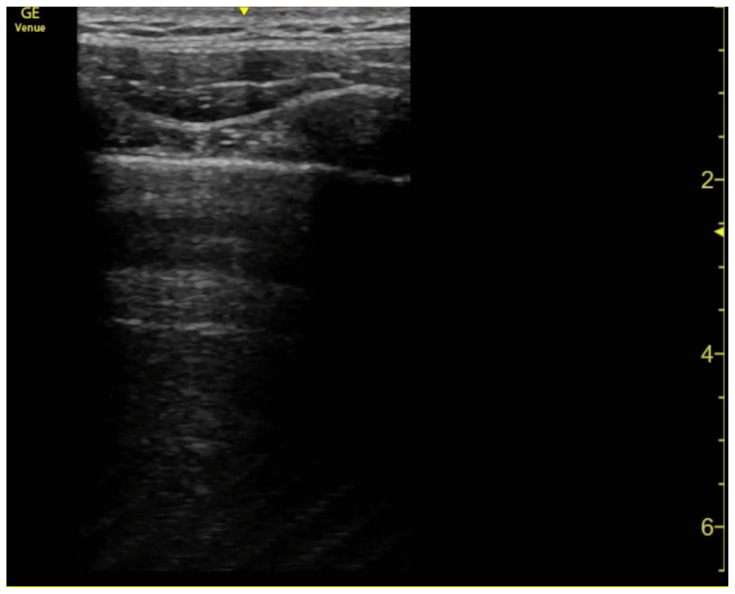


**Figure:**
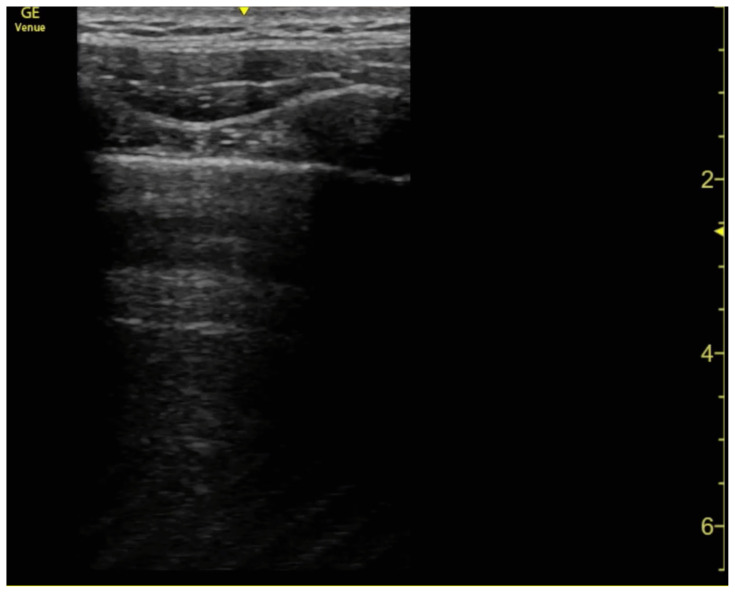


**Figure:**
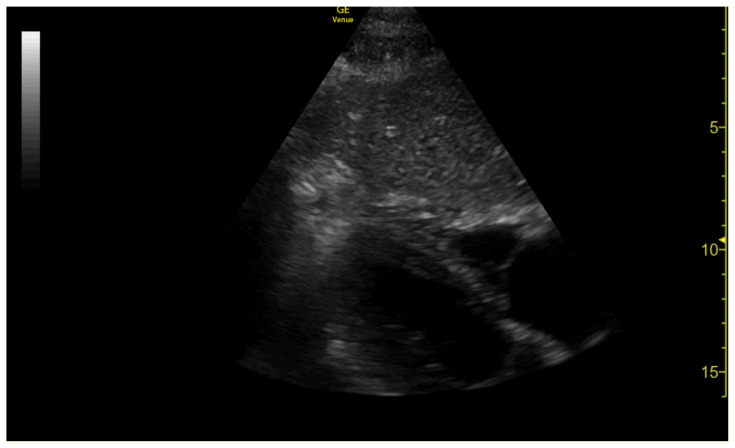


**Figure:**
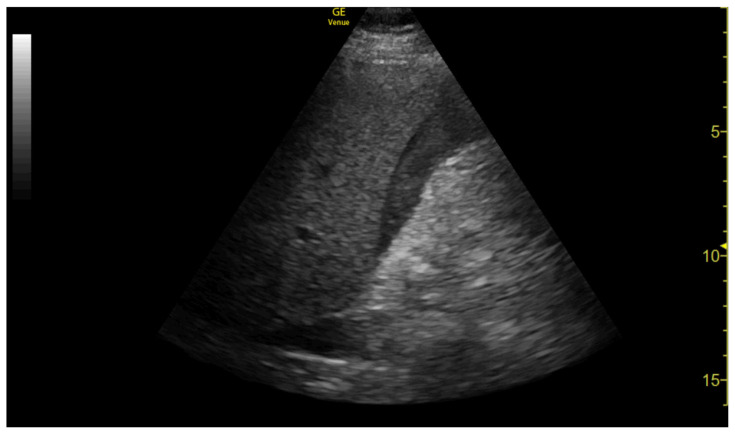


**Table:** SIMULATION EVENTS TABLE:

Minute (state)	Participant action/trigger	Patient status (simulator response) & operator prompts	Monitor display (vital signs)
6:00 (Baseline)		Six min after scenario start, this patient arrives regardless of team readiness. This patient has a GSW to the abdomen, to a leg with devastating injury vs amputation, and the opposite groin. Unconscious, groans to pain only, flexion of elbows to pain only, eyes remain closed.	T normal HR 160 BP 60/palp RR 45 O2 undetectable GCS 6 (E1 V2 M3) Bleeding from leg despite tourniquet. No distal pulse in the leg with the groin injury.
7:00 (One minute after this patient’s arrival).	No pressure on groin wound.	Rapid worsening of vital signs with progression to death.	HR 170 BP 40/palp RR 30 Rapid progression to PEA arrest
At any point during case.	Intubation with RSI.	Sats to low 90s (if blood pressure is >60/p, otherwise undetectable).	HR 160 BP 70/palp RR bagging O2 90%
At any point during case.	Release pressure on groin wound (hand/junctional tourniquet or wound packing).	Drop blood pressure and increase heart rate.	Temp 95 °F BP 40/palp HR 160 RR:40 O2 Sats 70% GCS 3 (E1, V1, M1)
	POCUS exam.	Positive abdominal free fluid.	
	Arrest state.	If the patient has been at the lowest point of the “death spiral” for 1 minute, he goes into a PEA arrest until 2 units of blood are transfused and pressure maintained on the groin wound.	
(Case Completion)	Physician decides on disposition or 10 minutes from start of case.	Move the patient out of the room. Assume any disposition option is ready for the patient.	

**Table:** 

**Critical Actions:** Place direct pressure over groin wound Place tourniquet on leg amputation if bleeding Establish IV or IO access Give RSI meds to facilitate intubation Intubate or place supraglottic airway Transfuse blood products per local protocol Give TXA Place pressure dressing over groin wound Decide disposition for patient	0:00

**Table:** 

	Milestone	Did not achieve level 1	Level 1	Level 2	Level 3
1	**Emergency Stabilization (PC1)**	□ Did not achieve Level 1	□ Recognizes abnormal vital signs	□ Recognizes an unstable patient, requiring intervention Performs primary assessment Discerns data to formulate a diagnostic impression/plan	□ Manages and prioritizes critical actions in a critically ill patient Reassesses after implementing a stabilizing intervention
2	**Performance of focused history and physical (PC2)**	□ Did not achieve Level 1	□ Performs a reliable, comprehensive history and physical exam	□ Performs and communicates a focused history and physical exam based on chief complaint and urgent issues	□ Prioritizes essential components of history and physical exam given dynamic circumstances
3	**Diagnostic studies (PC3)**	□ Did not achieve Level 1	□ Determines the necessity of diagnostic studies	□ Orders appropriate diagnostic studies. Performs appropriate bedside diagnostic studies/procedures	□ Prioritizes essential testing Interprets results of diagnostic studies Reviews risks, benefits, contraindications, and alternatives to a diagnostic study or procedure
4	**Diagnosis (PC4)**	□ Did not achieve Level 1	□ Considers a list of potential diagnoses	□ Considers an appropriate list of potential diagnosis May or may not make correct diagnosis	□ Makes the appropriate diagnosis Considers other potential diagnoses, avoiding premature closure
5	**Pharmacotherapy (PC5)**	□ Did not achieve Level 1	□ Asks patient for drug allergies	□ Selects an medication for therapeutic intervention, consider potential adverse effects	□ Selects the most appropriate medication and understands mechanism of action, effect, and potential side effects Considers and recognizes drug-drug interactions
6	**Observation and reassessment (PC6)**	□ Did not achieve Level 1	□ Reevaluates patient at least one time during case	□ Reevaluates patient after most therapeutic interventions	□ Consistently evaluates the effectiveness of therapies at appropriate intervals
7	**Disposition (PC7)**	□ Did not achieve Level 1	□ Appropriately selects whether to admit or discharge the patient	□ Appropriately selects whether to admit or discharge Involves the expertise of some of the appropriate specialists	□ Educates the patient appropriately about their disposition Assigns patient to an appropriate level of care (ICU/Tele/Floor) Involves expertise of all appropriate specialists
9	**General Approach to Procedures (PC9)**	□ Did not achieve Level 1	□ Identifies pertinent anatomy and physiology for a procedure Uses appropriate Universal Precautions	□ Obtains informed consent Knows indications, contraindications, anatomic landmarks, equipment, anesthetic and procedural technique, and potential complications for common ED procedures	□ Determines a back-up strategy if initial attempts are unsuccessful Correctly interprets results of diagnostic procedure
20	**Professional Values (PROF1)**	□ Did not achieve Level 1	□ Demonstrates caring, honest behavior	□ Exhibits compassion, respect, sensitivity and responsiveness	□ Develops alternative care plans when patients’ personal beliefs and decisions preclude standard care
22	**Patient centered communication (ICS1)**	□ Did not achieve level 1	□ Establishes rapport and demonstrates empathy to patient (and family) Listens effectively	□ Elicits patient’s reason for seeking health care	□ Manages patient expectations in a manner that minimizes potential for stress, conflict, and misunderstanding. Effectively communicates with vulnerable populations, (at risk patients and families)
23	**Team management (ICS2)**	□ Did not achieve level 1	□ Recognizes other members of the patient care team during case (nurse, techs)	□ Communicates pertinent information to other healthcare colleagues	□ Communicates a clear, succinct, and appropriate handoff with specialists and other colleagues Communicates effectively with ancillary staff

## INSTRUCTOR MATERIALS

**Case Title:** Small-Scale High-Fidelity Simulation for Mass Casualty Incident Readiness

**Case Description & Diagnosis (short synopsis):** Three patients are presented, each 3 minutes apart, with injuries consistent with penetrating trauma from a mass casualty incident. The team will need to perform life-saving measures such as intubation, chest decompression or chest tube placement, and wound management such as tourniquets in a timely fashion and disposition the patients as appropriate in the setting of an MCI.

Each simulation round has 3 patients. Patients A, B, and C are used in one round while patients D, E, and F are used in the other. To minimize time spent turning over the manikins, we suggest alternating the order of the patients. This is optional.

Patients have minimal physiologic changes because we had only one simulation operator programming all three cases. Only one of the three patients is able to speak to minimize confusion for the simulation operator.

**Equipment or Props Needed:**

❏ 3 high-fidelity simulators; 2 adults and 1 pediatric
❏ Gurney × 3
❏ Personal protective equipment
  ❏ Gowns, gloves, face masks, eye protection, bouffants or surgical caps as allowed per simulation center guidelines and supply availability
❏ Vascular access
  ❏ IV (intravenous) start supplies, IO (intraosseous) drill and needles, blood tubing, IV tubing, IV crystalloid bags, blood bags, blood bank tubes
❏ Medications
  ❏ Medication vials, syringes, needleless adapters (have nurses go through motion of injecting if not feasible to allow actual injection)
  ❏ Rapid sequence induction meds such as ketamine, etomidate, succinylcholine, rocuronium or what is anticipated to be available in an MCI
  ❏ Pain medications such as fentanyl, hydromorphone, morphine
  ❏ Tranexamic acid (TXA)
  ❏ IV fluids
  ❏ Simulated blood products
❏ Intubation equipment
  ❏ Laryngoscope handle and blade, suction, ET tube, stylet and/or endotracheal tube introducer/bougie, 10 mL syringe, stethoscope, lubricant for manikin, bag-valve-mask, supraglottic airway
❏ Chest tube equipment
  ❏ Simulator/part-task trainer if manikin cannot have chest cavity opened (for instance TraumaMan,R SIMULAB, Seattle, WA, United States).
  ❏ Chest tube insertion kits, 14g needles for decompression, supplies for securing (petrolatum gauze strips, gauze pads, tape), permanent ink marker (for writing on dressing), tubing and chest tube drainage system, cable ties, cable tie gun. Note: Remind learners not to write on the manikins with the marker.
  ❏ Tourniquets (3), hot hats, blankets, wound packing supplies (rolled gauze, any proprietary wound packing stocked by the facility for MCI usage), cervical collars, central line kits, crash carts, equipment for finger thoracostomy.
❏ Ultrasound machine (likely will not use on manikins but useful to have for logistical purposes).
❏ Printouts of representative ultrasound images (see stimulus inventory).

**Confederates needed:**

- EMS/Staff member to bring new patients in (exact role would depend on the hospital’s MCI plan).
- Attending physician - available to briefly answer questions and support decision-making by resident physician but should not offer hands-on care.
- Charge nurse or bed control nurse - primary role is to place added pressure to disposition patients quickly. If sufficient staffing is not available, an attending physician places this pressure on disposition.

**Stimulus Inventory:**

Appendix A: Simulation Events Table, Round 1

Appendix B: Simulation Events Table, Round 2

Appendix C: Critical Intake - MARCH Algorithm Poster

Appendix D: Task Completion Statistics

Appendix E: Case Stem

Appendix F: Supplies

Appendix G: Stimulus Inventory

Appendix H: Abbreviation Key

Appendix I: Participant Survey for Site 1

Appendix J: Participant Survey Results for Site 1

Appendix K: Post Simulation Participant Survey Results for Site 2

**Tech/Patient reset checklist**

1. Insure correct patient outline for each manikin: A, B, C, etc.
2. Move the wounds to the correct new location
3. Place clothing and cut wound holes as necessary per script
4. Douse with blood as necessary per script
5. Load arterial bleeders as necessary per script
6. Review script/outcomes

**Background and brief information:** The hospital receives reports of a mass shooting nearby and is expecting multiple patients with penetrating injuries. A mass casualty incident activation occurs. The OR has been notified. Multiple rooms and teams are prepared and ready to receive patients.

**Initial presentation:** There are 6 different patient presentations, with 3 patients per round; see initial presentation of each patient below.

**How the scene unfolds:** Each 10-minute simulation round consists of 3 patients with penetrating injuries, arriving at time 0, 3 minutes, and 6 minutes.

**Critical actions:**

Critical actions are listed with each patient description; see below.

*Small-Scale, High-Fidelity Simulation for Mass Casualty Readiness*

*Patient A – (Round 1, Patient 1)*

**Chief Complaint:** The hospital receives reports of a mass shooting nearby and is expecting multiple patients with penetrating injuries. A mass casualty incident activation occurs.

Absent lung sounds on wounded left side, thready pulses, absent pulse distal to right groin wound, barely awake--groans only, left pupil dilated.

**General Appearance:** left head wound, left chest wound, right groin wound (too high for tourniquet), bloody clothes, sweaty skin

**Primary Survey:**

- **Airway:** Normal, Negative (patent)
- **Breathing:** rapid and shallow, decreased breath sounds on left side with chest wound
- **Circulation:** thready radial pulses, thready femoral pulses (if asked, verbally note pulse to be absent in left foot)

**History:**

- **History of present illness:** involved in mass casualty incident with penetrating trauma
- **Past medical history, past surgical history, medications, allergies, social history, and family history**: unknown

**Secondary Survey/Physical Examination:**

- **General appearance:** critically injured, wearing clothes covered in blood over chest and groin
- **HEENT:**
  ○ **Head:** Left sided depressed circular wound with slow oozing
  ○ **Eyes:** Left pupil fixed and dilated, right pupil 4 millimeters and reactive
  ○ **Ears:** left hemotympanum
  ○ **Nose:** within normal limits
  ○ **Throat:** mild hemoptysis
- **Neck:** within normal limits
- **Heart:** tachycardic
- **Lungs:** absent lung sounds on left
- **Chest:** bullet wound left chest; right chest normal
- **Abdominal/GI:** normal (soft, nondistended, no grimace to palpation)
- **Genitourinary:** GSW to right groin--too high for tourniquet
- **Lower Extremities (legs):** right groin with deep wound with brisk bleeding, left normal (no deformities, normal passive range of motion)
- **Back:** within normal limits
- **Neuro:** GCS (Glasgow Coma Scale) 8; E2 V2 M4 (eyes, verbal, motor sub scores)
- **Skin:** cool, sweaty
- **Lymph:** within normal limits
- **Psych:** unable to assess

**Results:**

*No labs available in the time course of case*.

*US (ultrasound) images (show only if US done or asked for)*

Author’s own images

Left side of chest: US Image 4 Abnormal Lung Sliding
  https://youtu.be/fON9KaI2kC8
Right Side of chest: US Image 3 Normal Lung Sliding
  https://youtu.be/gHlg5m--3d0
Subxiphoid view: US Image 5 Normal subxiphoid cardiac view
RUQ view: US Image 1 Normal RUQ

## OPERATOR MATERIALS

*Small-Scale, High-Fidelity Simulation for Mass Casualty Readiness*

*Patient A – (Round 1, Patient 1)*

**Diagnosis:**

GSW to head, chest, groin

**Disposition:**

Operating room

## SIMULATION ASSESSMENT

*Small-Scale, High-Fidelity Simulation for Mass Casualty Readiness*

*Patient A – (Round 1, Patient 1)*

Learner: _________________________________________

***Assessment Timeline***

This timeline is to help observers assess their learners. It allows observer to make notes on when learners performed various tasks, which can help guide debriefing discussion.

**Critical Actions:**

□ Place direct pressure over groin wound
□ Establish IV or IO access
□ Give RSI (rapid sequence induction) meds to facilitate intubation
□ Intubate or place supraglottic airway
□ Decompress chest on the left side of the GSW
□ Transfuse blood products per local protocol
□ Give TXA (Tranexamic acid)
□ Place pressure dressing over groin wound
□ Decide disposition for patient

**Summative and formative comments:**

**Milestones assessment:**

## INSTRUCTOR MATERIALS

*Small-Scale, High-Fidelity Simulation for Mass Casualty Readiness*

*Patient B – (Round 1, Patient 2)*

**Chief Complaint:** Thirty-two y/o adult with GSW to right thoracoabdominal region, GSW left leg (amputation)

screaming about pain in extremity and demanding status report on “my baby”

**General Appearance:** Loud and awake, confused and screaming, GSW to right thoracoabdominal region. High velocity GSW that resulted in near amputation of left leg. Bloody clothes

**Primary Survey:**

- **Airway:** Normal, Negative (patent)
- **Breathing:** tachypneic, decreased on right side, normal breath sounds on left
- **Circulation:** 2 + radial pulses bilaterally (if they ask: left lower extremity amputation at the thigh with pulsatile bleeding, right leg with normal pulses)

**History:**

- **History of present illness:** involved in mass casualty incident with penetrating trauma. Pt screaming, “I can’t breathe… My baby! Where’s my baby?”
- **Past medical history, past surgical history, medications, allergies, social history, and family history**: patient not answering

**Secondary Survey/Physical Examination:**

- **General appearance:** distressed, loud and awake, confused and screaming. GSW to the right thoracoabdominal region. Amputated left leg. Dyspneic, trying to yell, asking about the location of their child
- **HEENT:**
  ○ **Head:** within normal limits
  ○ **Eyes:** within normal limits
  ○ **Ears:** within normal limits
  ○ **Nose:** within normal limits
  ○ **Throat:** within normal limits
- **Neck:** within normal limits
- **Heart:** tachycardic
- **Lungs:** decreased on right side of GSW
- **Abdominal/GI:** right thoracoabdominal GSW ×1, screams when right abdomen palpated, left side nontender
- **Genitourinary:** within normal limits
- **Extremities:** left lower extremity with deep wound and amputation (depending on manikin capability), right normal (no deformities, normal passive range of motion)
- **Back:** within normal limits
- **Neuro:** agitated but alert. GCS 15 (eyes 4, verbal 5, movement 6)
- **Skin:** cool, sweaty
- **Lymph:** within normal limits
- **Psych:** appropriately agitated

**Results:**

*No labs available in the time course of case*.

*US (ultrasound) images (show only if US done or asked for)*

Author’s own images

Left side of chest: US Image 3 Normal Lung Sliding
  https://youtu.be/gHlg5m--3d0
Right Side of chest: US Image 4 Abnormal Lung Sliding
  https://youtu.be/fON9KaI2kC8
Subxiphoid view: US Image 5 Normal subxiphoid cardiac view
RUQ view: US Image 2 RUQ positive for free fluid

## OPERATOR MATERIALS

*Small-Scale, High-Fidelity Simulation for Mass Casualty Readiness*

*Patient B – (Round 1, Patient 2)*

**Diagnosis:**

GSW to left leg with amputation/mangled extremity, GSW to right thoracoabdominal region

**Disposition:**

Operating room

## SIMULATION ASSESSMENT

*Small-Scale, High-Fidelity Simulation for Mass Casualty Readiness*

*Patient B – (Round 1, Patient 2)*

Learner: _________________________________________

***Assessment Timeline***

This timeline is to help observers assess their learners. It allows observer to make notes on when learners performed various tasks, which can help guide debriefing discussion.

**Critical Actions:**

□ Adjust tourniquet on leg to stop blood flow at site of amputation
□ Establish IV or IO access
□ Give RSI meds to facilitate intubation
□ Intubate or place supraglottic airway
□ Decompress chest on the right side of the GSW
□ Perform point-of-care ultrasound (POCUS) examination
□ Transfuse blood products per local protocol
□ Give TXA
□ Decide disposition for patient

**Summative and formative comments:**

**Milestones assessment:**

## INSTRUCTOR MATERIALS

*Small-Scale, High-Fidelity Simulation for Mass Casualty Readiness*

*Patient C – (Round 1, Patient 3)*

**Chief Complaint:** Child (age determined by manikin availability), female with Injuries: GSW right neck, GSW right abdomen

thready central pulses only, absent distal pulses, unresponsive agonal respirations

**General Appearance:** critically ill child, large volume hemorrhage around neck, GSW to neck and abdomen

**Primary Survey:**

- **Airway:** not protected, blood coming from airway
- **Breathing:** agonal
- **Circulation:** weak carotid pulses, no radial pulses, no femoral pulses

**History:**

- **History of present illness:** involved in mass casualty incident with penetrating trauma, unable to provide further history
- **Past medical history, past surgical history, medications, allergies, social history, and family history**: unknown

**Secondary Survey/Physical Examination:**

- **General appearance:** critically ill, in extremis
- **HEENT:**
  ○ **Head:** within normal limits
  ○ **Eyes:** pupils 6mm and non-reactive
  ○ **Ears:** within normal limits
  ○ **Nose:** within normal limits
  ○ **Throat:** within normal limits
- **Neck:** GSW with large volume surrounding blood to right side
- **Heart:** bradycardic
- **Lungs:** minimal lung sounds due to agonal breathing. If airway secured, normal/symmetric.
- **Abdominal/GI:** Bullet wound but no grimace to palpation, abdomen firm
- **Genitourinary:** within normal limits
- **Rectal:** within normal limits
- **Extremities:** within normal limits
- **Back:** within normal limits
- **Neuro:** GCS 3
- **Skin:** cool and sweaty, pale/gray
- **Lymph:** within normal limits
- **Psych:** unable to assess

**Results:**

*No labs available in the time course of case*.

*US (ultrasound) images (show only if US done or asked for)*

Author’s own images

Left side of chest: US Image 3 Normal Lung Sliding
  https://youtu.be/gHlg5m--3d0
Right Side of chest: US Image 3 Normal Lung Sliding
  https://youtu.be/gHlg5m--3d0
Subxiphoid view: US Image 5 Normal subxiphoid cardiac view
RUQ view: US Image 2 RUQ positive for free fluid

## OPERATOR MATERIALS

*Small-Scale, High-Fidelity Simulation for Mass Casualty Readiness*

*Patient C – (Round 1, Patient 3)*

**Diagnosis:**

GSW to neck and abdomen

**Disposition:**

Deceased/morgue (regardless of learner intervention, this patient dies)

## SIMULATION ASSESSMENT

*Small-Scale, High-Fidelity Simulation for Mass Casualty Readiness*

*Patient C – (Round 1, Patient 3)*

Learner: _________________________________________

***Assessment Timeline***

This timeline is to help observers assess their learners. It allows observer to make notes on when learners performed various tasks, which can help guide debriefing discussion.

**Critical Actions:**

□ Place direct pressure on neck wound
□ Establish IV or IO access
□ Perform POCUS exam
□ Intubate or place supraglottic airway
□ Stop resuscitative efforts and transfer to areas for expectant care or deceased patients

**Summative and formative comments:**

**Milestones assessment:**

## INSTRUCTOR MATERIALS

*Small-Scale, High-Fidelity Simulation for Mass Casualty Readiness*

*Patient D – (Round 2, Patient 1)*

**Chief Complaint:** Fifty-four y/o Male with injuries: GSW to neck, GSW to chest

absent lung sounds chest wound site, thready pulses, barely awake, groans only

**General Appearance:** GSW to neck and chest

**Primary Survey:**

- **Airway:** Normal, Negative (patent)
- **Breathing:** absent lung sounds on left side of GSW
- **Circulation:** thready radial pulses, thready femoral pulses

**History:**

- **History of present illness:** involved in mass casualty incident with penetrating trauma
- **Past medical history, past surgical history, medications, allergies, social history, and family history**: unknown

**Secondary Survey/Physical Examination:**

- **General appearance:** critically ill, GSW to right side neck and left chest, lots of blood
- **HEENT:**
  ○ **Head:** within normal limits
  ○ **Eyes:** sluggish bilaterally.
  ○ **Ears:** within normal limits
  ○ **Nose:** within normal limits
  ○ **Throat:** within normal limits
- **Neck:** GSW to right side of neck, profuse hemorrhage
- **Heart:** tachycardic
- **Lungs:** absent on left side of GSW
- **Abdominal/GI:** within normal limits
- **Genitourinary:** within normal limits
- **Rectal:** within normal limits
- **Extremities:** within normal limits
- **Back:** within normal limits
- **Neuro:** GCS 4 (moaning, eyes closed, not moving)
- **Skin:** cool and sweaty, pale
- **Lymph:** within normal limits
- **Psych:** unable to assess

**Results:**

*No labs available in the time course of case*.

*US (ultrasound) images (show only if US done or asked for)*

Author’s own images

Left side of chest: US Image 4 Abnormal Lung Sliding
  https://youtu.be/fON9KaI2kC8
Right Side of chest: US Image 3 Normal Lung Sliding
  https://youtu.be/gHlg5m--3d0
Subxiphoid view: US Image 5 Normal subxiphoid cardiac view
RUQ view: US Image 1 Normal RUQ

## OPERATOR MATERIALS

*Small-Scale, High-Fidelity Simulation for Mass Casualty Readiness*

*Patient D – (Round 2, Patient 1)*

**Diagnosis:**

GSW to neck and chest

**Disposition:**

Operating room

## SIMULATION ASSESSMENT

*Small-Scale, High-Fidelity Simulation for Mass Casualty Readiness*

*Patient D – (Round 2, Patient 1)*

Learner: _________________________________________

***Assessment Timeline***

This timeline is to help observers assess their learners. It allows observer to make notes on when learners performed various tasks, which can help guide debriefing discussion.

**Critical Actions:**

□ Place direct pressure over neck wound
□ Establish IV or IO access
□ Give RSI meds to facilitate intubation
□ Intubate or place supraglottic airway
□ Decompress chest on the side of the GSW
□ Transfuse blood products per local protocol
□ Give TXA
□ Place pressure dressing over neck wound
□ Decide disposition for patient

**Summative and formative comments:**

**Milestones assessment:**

## INSTRUCTOR MATERIALS

*Small-Scale, High-Fidelity Simulation for Mass Casualty Readiness*

*Patient E – (Round 2, Patient 2)*

**Chief Complaint:** Eighteen y/o female injuries: GSW to left head, GSW right thoracoabdominal region

absent lung sounds on wounded right side, thready pulses, barely awake, groans only, left pupil dilated (only tell if asked or directly examined if manikin can’t do this)

**General Appearance:** GSW to head and thoracoabdominal region, bloody clothes, sweaty skin

**Primary Survey:**

- **Airway:** not adequately protected, no blood visualized
- **Breathing:** absent breath sounds on right
- **Circulation:** thready radial pulses, thready femoral pulses

**History:**

- **History of present illness:** involved in mass casualty incident with penetrating trauma
- Past medical history, past surgical history, medications, allergies, social history, and family history: unknown

**Secondary Survey/Physical Examination:**

- **General appearance:** critically ill appearing with GSW to head and thoracoabdominal region
- **HEENT:**
  ○ **Head:** left side with depressed circular wound with slow oozing; right side normal
  ○ **Eyes:** GSW left side, left pupil fixed and dilated; right side pupil 4 millimeters and reactive bilaterally sluggish
  ○ **Ears:** left hemotympanum
  ○ **Nose:** within normal limits
  ○ **Throat:** within normal limits
- **Neck:** within normal limits
- **Heart:** tachycardic
- **Lungs:** absent on right
- **Abdominal/GI:** bullet wound--groans with palpation
- **Genitourinary:** within normal limits
- **Rectal:** within normal limits
- **Extremities:** within normal limits
- **Back:** within normal limits
- **Neuro:** GCS 10 (eyes 3, verbal 2, movement 5), moaning
- **Skin:** cool and sweaty
- **Lymph:** within normal limits
- **Psych:** unable to assess

**Results:**

*No labs available in the time course of case*.

*US (ultrasound) images (show only if US done or asked for)*

Author’s own images

Left side of chest: US Image 3 Normal Lung Sliding
  https://youtu.be/gHlg5m--3d0
Right Side of chest: US Image 4 Abnormal Lung Sliding
  https://youtu.be/fON9KaI2kC8
Subxiphoid view: US Image 5 Normal subxiphoid cardiac view
RUQ view: US Image 2 RUQ positive for free fluid

## OPERATOR MATERIALS

*Small-Scale, High-Fidelity Simulation for Mass Casualty Readiness*

*Patient E – (Round 2, Patient 2)*

**Diagnosis:**

GSW to head and thoracoabdominal region

**Disposition:**

Operating room

## SIMULATION ASSESSMENT

*Small-Scale, High-Fidelity Simulation for Mass Casualty Readiness*

*Patient E – (Round 2, Patient 2)*

Learner: _________________________________________

***Assessment Timeline***

This timeline is to help observers assess their learners. It allows observer to make notes on when learners performed various tasks, which can help guide debriefing discussion.

**Critical Actions:**

□ Establish IV or IO access
□ Give RSI meds to facilitate intubation
□ Intubate or place supraglottic airway
□ Decompress chest on the right side of the GSW
□ Transfuse blood products per local protocol
□ Give TXA
□ Decide disposition for patient

**Summative and formative comments:**

**Milestones assessment:**

## INSTRUCTOR MATERIALS

*Small-Scale, High-Fidelity Simulation for Mass Casualty Readiness*

*Patient F – (Round 2, Patient 3)*

**Chief Complaint:** Twenty-three y/o with injuries: GSW right side of abdomen, GSW left leg (amputation), GSW to right groin (opposite leg injury)

thready pulses and no distal pulses to leg wound/amputation, barely awake--groans only

**General Appearance:** GSW to abdomen, GSW to groin, GSW to contralateral leg with amputation

**Primary Survey:**

- **Airway:** moaning, not adequately protecting
- **Breathing:** Normal (clear and equal bilaterally)
- **Circulation:** 2+ radial pulses bilaterally (if asked: left lower extremity amputation at the thigh and pulsatile bleeding, right side with absent pulses)

**History:**

- **History of present illness:** involved in mass casualty incident with penetrating trauma
- **Past medical history, past surgical history, medications, allergies, social history, and family history**: unknown

**Secondary Survey/Physical Examination:**

- **General appearance:** critically ill appearing with GSW to right abdomen, left leg with amputation or mangling (based on manikin capability), and contralateral groin
- **HEENT:**
  ○ **Head:** within normal limits
  ○ **Eyes:** within normal limits
  ○ **Ears:** within normal limits
  ○ **Nose:** within normal limits
  ○ **Throat:** within normal limits
- **Neck:** within normal limits
- **Heart:** tachycardic
- **Lungs:** tachypneic, symmetric
- **Abdominal/GI:** bullet wound--groans when abdomen is touched on right side
- **Genitourinary:** within normal limits
- **Rectal:** within normal limits
- **Extremities:** left lower extremity with deep wound and amputation (depending on manikin capability); right groin with deep wound with brisk bleeding traumatic amputation or mangling of one leg
- **Back:** within normal limits
- **Neuro:** GCS 9 (eyes 2, verbal 2, movement 5), moaning
- **Skin:** cool, pale, diaphoretic
- **Lymph:** within normal limits
- **Psych:** unable to assess

**Results:**

*No labs available in the time course of case*.

*US (ultrasound) images (show only if US done or asked for)*

Author’s own images

Left side of chest: US Image 3 Normal Lung Sliding
  https://youtu.be/gHlg5m--3d0
Right Side of chest: US Image 3 Normal Lung Sliding
  https://youtu.be/gHlg5m--3d0
Subxiphoid view: US Image 5 Normal subxiphoid cardiac view
RUQ view: US Image 2 RUQ positive for free fluid

## OPERATOR MATERIALS

*Small-Scale, High-Fidelity Simulation for Mass Casualty Readiness*

*Patient F – (Round 2, Patient 2)*

**Diagnosis:**

GSW to abdomen, GSW to groin, GSW to contralateral leg with amputation

**Disposition:**

Operating room

## SIMULATION ASSESSMENT

*Small-Scale, High-Fidelity Simulation for Mass Casualty Readiness*

*Patient F – (Round 2, Patient 3)*

Learner: _________________________________________

***Assessment Timeline***

This timeline is to help observers assess their learners. It allows observer to make notes on when learners performed various tasks, which can help guide debriefing discussion.

**Critical Actions:**

□ Place direct pressure over groin wound
□ Place tourniquet on leg amputation if bleeding
□ Establish IV or IO access
□ Give RSI meds to facilitate intubation
□ Intubate or place supraglottic airway
□ Transfuse blood products per local protocol
□ Give TXA
□ Place pressure dressing over groin wound
□ Decide disposition for patient

**Summative and formative comments:**

**Milestones assessment:**

## DEBRIEFING AND EVALUATION PEARLS

### Mass Casualty Incident (MCI)

A Mass Casualty incident (MCI) is defined as “an event that overwhelms the local healthcare system, where the number of casualties vastly exceeds the local resources and capabilities in a short period of time.”^1^ Any MCI can rapidly exhaust available resources for not only the MCI but the normal day-to-day tasks for the hospital.

Crisis standard of care is implemented when standard contingency plans for a surge in demand for care are insufficient. The surge capacity following a mass casualty incident falls into three basic categories depending on the magnitude of the event: conventional, contingency, and crisis surge capacity. The categories will also represent a corresponding spectrum of patient care delivered during a disaster event.^2^

Incident planning and response must recognize that the standard of care may change and protocols for triage will need to be adaptable. Along the spectrum of care, there may be patients who are ‘too well’ to receive care and some who are too sick to survive but all will need to receive some level of health care consideration.^3^, ^4^

**Other debriefing points:** Debriefing was done by a panel of faculty who had observed but not participated in the simulation.^5^ Emergency medicine attending, trauma surgeon, trauma nurse educator were used in our settings. Participants were debriefed in a separate room with time allowed to decompress prior to defining and deepening learning objectives.

Collaboration with other services such as ancillary staff, trauma surgery, and other services that are outside the core of the ED staff can be halted by lack of communication due to infrequent prior contact. This is also improved in the debriefing afterward as everyone states their concerns and works with different points of view.

While the simulation was perceived as stressful, feedback from residents was very positive (see Appendix K). This simulation is meant to recreate the pressure an Emergency Physician will face if confronted with a mass shooting, such as the many such instances in the United States in recent years. This is a nearly impossible scenario with multiple critically injured patients presenting nearly simultaneously. During implementation, we referred to this case as the Kobayashi Maru from Star Trek fame.^6^ We forced residents to make hard decisions such as stopping resuscitative efforts in patients who might have survived if we could devote more resources to them.

This simulation was very hard on participants as well as observers. It is vital to let the learners talk through the emotions they face during and after this case. An experienced debriefer is vital.

Pre-briefing is critical. Let them know they will face a multiple patient encounter and will need to make very hard decisions. Make sure they know they can ask the attending for advice and cognitive backup whenever needed. During implementation, several residents asked for assistance when faced with stopping resuscitation efforts on patients, particularly the pediatric patient.

If not able to have all learners participate in the team leader role, some careful consideration to team leader selection is key. If there are learners with particular experience in this area (tactical EMS, military, prior relevant personal experience), we recommend not having those people directly participate. While any simulation can trigger an emotional response, this scenario is particularly high-risk for triggering unpleasant memories and experiences. They may or may not want to watch and should be excused if they wish. Alternatively, they may not struggle nearly as much as a learner without this prior training. A different learner may stand to gain much more in the simulation and the experienced learner can provide excellent feedback in the debriefing.

Work with the learners to determine which actions are truly needed. We quickly walked through one patient and had the participants list what actions they were taking. The debriefer can ask why that specific action is needed. Observers and participants can discuss what information is gathered from each intervention and whether that information can be omitted or gleaned from other sources (for instance, the cardiac monitor can be deferred for pulse oximetry or frequent pulse checks).

Make sure someone is taking notes since the participants and observers will likely come up with excellent ideas that can be incorporated into disaster planning.

**Wrap Up:** The participants were encouraged to contemplate their roles in the simulation and consider how they could apply this to a future clinical scenario. Potential barriers to implementation of a disaster plan were reviewed. Participant feedback was solicited for fidelity and relevance of the scenario. Wellness resources were offered, if desired. We recommend publicly thanking the simulation staff involved because this scenario is both logistically and emotionally challenging.

## INSTRUCTOR MATERIALS

### Appendix A Simulation Events Table, Round 1

**Table:**

Round 1			
Patients	**Patient A** Adult or Child Injuries: 1-GSW left head (one side only) 3-GSW left chest 6-GSW Right groin Procedures needed: Direct pressure on groin wound Wound packing Intubation Chest decompression Autotransfusion would be nice IV/IO x 2 TXA can be given but should not delay OR	**Patient B** Adult Injuries: 4-GSW Right thoracoabdomen 7-GSW Left leg with amp Procedures needed: Chest decompression Maybe intubation IV/IO x 2 TXA can be given but should not delay OR	**Patient C** Child Injuries: 2-GSW Right neck 5-GSW Right abd Procedures needed: patient crashes at a certain point - no CPR
Initial Manikin Setup	Wounds on head, one side of chest (clearly in anterior chest), one groin (too high for tourniquet), lots of bloody clothes/dressing at groin, cool, sweaty skin Background VS: 60/p, HR 140 Sats undetectable. Absent lung sounds on the side of the shot chest. Thready distal pulses all over, but absent distal pulse on leg on side of groin injury. Barely awake – just groaning	Wounds on chest/abd junction Amputated bleeding leg (add a poorly positioned tourniquet that is not working). Screaming patient. “I can’t ...breathe!” “My...leg.” “My...baby.” “Where’s...my...baby?” VS: 70/p, HR 140, sats in 80s	Lots of blood around the neck. Wounds at neck and belly No distal pulses (BP 40 or so) Thready central pulses No voice, unresponsive
Response to Treatment	No pressure on groin wound within 1 min, start rapid “death spiral” Release pressure on groin wound – drop BP and increase HR Chest decompression increases BP to 90/p and drops HR to 110	Tightening, replacing or supplementing tourniquet will stop bleeding After chest decompression, breathing, HR better, BP up to 80s. Blood or IV fluids get BP to 90s. Loud and distracting until sedated or intubated	Will die no matter what
0–0:30sec	Arrives		
0:31–1:00			
1:01–1:30			
1:31–2:00			
2:01–2:30			
2:31–3:00			
3:01–3:30		Arrives – yelling, very dramatic	
3:31–4:00			
4:01–4:30			
4:31–5:00			
5:01–5:30			
5:31–6:00			
6:01–6:30			Arrives
6:31–7:00			
7:01–7:30			
7:31–8:00			Pulses go away (PEA arrest)
8:01–8:30			
9:00–9:30			
9:31–10:00			
10:00	Scenario ends		

### Appendix B Simulation Events Table, Round 2

**Table:**

Round 2			
Patients	**Patient D** Adult or Child Manikin Injuries: 2-GSW right neck – either side 3-GSW left chest Procedures needed: Direct pressure on neck Intubation Chest decompression Autotransfusion can be considered IV/IO x 2 TXA can be given but should not delay OR	**Patient E** Adult or Child Injuries: 1-GSW left head (one side only) 4-GSW right thoracoabdomen Procedures needed: Chest decompression IV/IO x 2 TXA can be given but should not delay OR	**Patient F** Adult or Child Injuries: 5-GSW right abd 7-GSW left leg with amp 6-GSW right groin (opposite side as leg amp) Procedures needed: Tourniquet placement IV/IO x 2 Direct pressure on groin wound Wound packing TXA can be given but should not delay OR
Initial Manikin Setup	Wounds as above Lots of blood around neck Cool, sweaty skin Background VS: 60/p, HR 140, sats undetectable Absent lung sounds on what side of the chest was shot Barely awake – just groaning	Wounds on head and chest/abd junction Cool, sweaty skin VS: 70/p, HR 140	Wounds abd, leg, groin Amputated bleeding leg with an insufficient tourniquet Cool, sweaty skin VS: BP 60/p, HR 160 No distal pulses all over until resuscitated, but absent distal pulse on leg on side of groin injury.
Response to Treatment	No pressure at neck within 1 min? Start rapid progression to death Release neck pressure? Start dropping BP and increasing HR	Chest tube improves VS	No pressure on groin wound within 1 min, start rapid progression to death spiral Release pressure on groin wound – drop BP and increase RH
0–0:30sec	Arrives		
0:31–1:00			
1:01–1:30			
1:31–2:00			
2:01–2:30			
2:31–3:00			
3:01–3:30		Arrives	
3:31–4:00			
4:01–4:30			
4:31–5:00			
5:01–5:30			
5:31–6:00			
6:01–6:30			Arrives
6:31–7:00			
7:01–7:30			
7:31–8:00			
8:01–8:30			
9:00–9:30			
9:31–10:00			
10:00	Scenario ends		

### Appendix D Task Completion Statistics

**Table:**

Round	1			2			3			4			5			6		
Team Leader	Group 1, G2 #1			Group 1, G2 #2			Group 2, G2 #3			Group 2, G2 #4			Group 3, G2 #5			Group 3, G2 #6		
Patient	A	B	C	D	E	F	D	E	F	A	B	C	A	B	C	D	E	F
Non-Physician Tasks																		
Direct Pressure	X	X		X	X		X	X		X			X		X	X	X	X
Tourniquet		X		X					X		X			X				X
Pressure Dressing				X			X	X	X	X			X	X		X	X	
Exposure	X	X				X	X									X		X
IV Established	X	X		X	X		X	X	X	X		X	X	X	X	X	X	X
Lab Drawn				X			X											
Fluids Given	X																	
TXA Given									X				X					X
Blood Given	X						X	X					X			X		
Bagging Patient	X	X		X	X		X	X	X	X				X		X		X
Ventilator Initiated	X																	
Supplemental O2									X									
C-collar Placed															X			
Patient to OR										X			X			X		X
Non-Physician Tasks Completed	7	5	0	6	3	1	7	5	6	5	1	1	6	4	3	7	3	7
Non-Physician Tasks Completed/Round	12			10			18			7			13			17		
Physician Tasks																		
Needle Thoracostomy	X						X			X								
Finger Thoracostomy	X				X													
Chest Tube				X			X			X	X		X	X				X
Intubation		/		X	X		X	/		X			X	/		X		X
Ultrasound	X													X				
Pronounced/Expectant			X			X						X			X		X	
Delay d/t Equipment Need or Failure																		
Resident Requested Staff to Pronounce												X			X			
Physician Tasks Completed	3	0.5	1	2	2	1	3	0.5	0	3	1	2	2	2.5	2	1	1	2
Physician Tasks Completed/Round	4.5			5			3.5			5			6.5			4		

**Table:**

Key	
X	Completed Task
/	Initiated task but did not complete
G2	EM resident PGY-2

Order of cases changed for rounds 3 and 4 only to make transitions easier for simulation staff

### Appendix E Case Stem

Welcome to Trauma room 3! It is your day off but you have been called in to help with an MCI. There is an active shooter scenario at a local scrubs factory. There are already 12 confirmed dead and the estimated injured are 4 to 5 times that. Trauma room one is full. Trauma room 2 is full. The next patients are coming to you! We have set up a separate room and are naming it trauma room three.

**The ‘Rules’**

1. What we do in the sim lab is for the patients. Yes, it's weird and artificial but I ask that you suspend your disbelief for the sake of practice. I believe that what you practice in the sim lab will transfer, in terms of experience and motor memory, to the bedside.
2. What happens in the sim lab, stays in the sim lab: Like Las Vegas, there may be suboptimal events that happen in the sim lab. I ask that you not judge nor gossip about those events. This is a judgment free zone. In healthcare, not everything goes according to plan. I think it’s safe to say we all prefer those unexpected actions and outcomes happen here, so we all can learn and grow from them, instead of in a real patient care scenario. Simulation is about creating a shared experience that we can talk about together and learn from. I don’t expect everything to happen perfectly in that room... far from it. We have fewer opportunities to learn from when everything goes like clockwork. Do not share the details of the case with other folks who might go through it in the future. They want to learn too!
3. Treat the manikin as you would a patient:
  - Practice makes Perfect!
  - CPR can be done just as in real life PLUS you can get feedback in real time
  - If you intubate, do so carefully: his skin can be torn
  - If you defibrillate, ask for help if unsure: it’s a real defibrillator!
  - Like charting: it's not done until you actually do it. No IV unless one is put in
  - If it's not clear (plastic skin, abnormal abdominal exam) ask, and we’ll clarify
  - *IV’s: The manikins in this exercise will come in with rubbery square patches over one antecubital fossa. There is a “vein” hidden inside that can actually be poked and an IV placed. Then fluids and meds can actually be given
  - There will also be simulated medication, blood products, and IV fluids. They should be given like in real life (although you can just trickle the drips so the reservoir bag doesn't fill up)
  - ** IO’s: if you can't start an IV, you can ask the doctor for an IO. They will drill one into a simulated tibia. Doctors, please use the tibia in the kits, not the actual manikin leg. This might vary as several manikin legs allow IO access.

### Appendix F Supplies

❏ Crash cart
❏ Chest tube kits
❏ Central line kits
❏ Tourniquet
❏ C- collars
❏ IO drill**: for an IO, drill one into a simulated tibia. Please use the simulated tibia in the kit, not the actual manikin leg
❏ For the monitors: Place the cables on the chest; then call out, “they are on the monitor!” For BP, just put cuff on the arm, “Taking a blood pressure now,” and put the loose finger probe on, “They are on saturation monitor.” The display will then magically show the readings
❏ You have IV start supplies, so please try the fake veins in the little plastic squares. The instructor will then turn the stopcock so you can give meds
❏ There are simulated meds: the patient needs to get a dose of these before “it counts”
❏ Only one ventilator. But if you put someone on the vent, an invisible RT will come magically to the side and the vent will be then on autopilot

### Appendix G Stimulus Inventory

**Table:**

Video	Format	Finding	Use for patients
Image 1	JPG	Normal RUQ (Authors’ own image)	A, D
Image 2	JPG	RUQ positive for free fluid (Authors’ own image)	B, C, E, F
Image 3	MP4	normal lung sliding (Authors’ own image)	All
Image 4	MP4	abnormal lung sliding (Authors’ own image)	A, B, C, D, E
Image 5	JPG	Normal subxiphoid cardiac view (Authors’ own image)	All

- US Image 1 normal RUQ: Authors’ Own Image
  Patients A, D
- US Image 2 RUQ positive for free fluid: Authors’ Own Image
  Patients B, C, E, F
- US Image 3 Normal lung sliding: Authors’ Own Image
  All Patients
  https://youtu.be/gHlg5m--3d0
- US Image 4 Abnormal lung sliding: Authors’ Own Image
  Patients A, B, C, D, E
  https://youtu.be/fON9KaI2kC8
- US Image 5 Subxiphoid normal: Authors’ Own Image
  Patients A, B, C, D, E, F

### Appendix H Abbreviation Key

- BP: Blood pressure
- POCUS: point-of-care ultrasound
- GCS: Glasgow Coma Scale
- GSW: Gunshot wound
- HR: Heart rate
- IV: Intravenous
- IO: Intraosseous
- RSI: Rapid sequence induction
- RR: Respiratory rate
- T: Temperature
- TXA: Tranexamic acid
- O_2_ Sat: Oxygen Saturation

### Appendix I Participant Survey for Site 1

**Level of training (circle):** G1 G2 G3 Staff Other: ___________________

Q1: I understand how patient flow through the ED would ideally work in a mass casualty incident.

○ Strongly disagree
○ Disagree
○ Neither agree nor disagree
○ Agree
○ Strongly agree

Q2: A Mass Casualty Incident (MCI) has happened. Region’s ED has activated its MCI protocol. Mass shooting with hundreds of victims. The Pod E staff physician directs the ED staff to transition to a “critical intake” model to expedite life-saving care of multiple critically injured patients. I understand what “critical intake” is.

○ Strongly disagree
○ Disagree
○ Neither agree nor disagree
○ Agree
○ Strongly agree

Q3: I feel comfortable being team lead in an MCI.

○ Strongly disagree
○ Disagree
○ Neither agree nor disagree
○ Agree
○ Strongly agree

Q4: Red tagged patient goes through Critical Intake. Which is true regarding labs in the Critical Intake phase of an MCI?

○ None will be drawn
○ Type and screen will be the only lab drawn
○ Stable Trauma Panel to be done on all patients as soon as possible in Critical Intake

Q5: According to Critical Intake guidelines, how would patients be monitored during an MCI?

○ Usual cardiac, BP, SpO2, +/− ETCO2 as needed
○ SpO2 only
○ Cardiac monitor only

Q6: During an MCI from a shooting, what imaging would be most useful and feasible during the initial 30–60 minutes of the incident when most critically injured patients are expected to arrive?

○ No imaging
○ POC US (extended FAST) only
○ POC US (extended FAST) + portable CXR
○ Usual imaging options as in a Triage and Treatment Area (portable chest +/− pelvis XR, extended FAST, CT as needed)

### Appendix J Participant Survey Results for Site 1

*Identical survey was given to participants before and after the simulation*.

Subjective questions (Q1–Q3)

**Table:**

		Strongly Disagree	Disagree	Neither Agree nor Disagree	Agree	Strongly Agree
Q1	Pre-Sim (n=24)	0	4	7	13	0
	Post-Sim (n=10)	0	0	0	8	2
Q2	Pre-Sim (n=23)	0	5	8	10	0
	Post-Sim (n=10)	0	0	0	9	1
Q3	Pre-Sim (n=24)	9	6	4	5	0
	Post-Sim (n=10)	1	0	6	4	0

Knowledge questions (Q4–Q6)

**Table:**

	Percentage Correct
Q4 Pre	10/24 (41.7%)
Q4 Post	7/10 (70%)
Q5 Pre	16/23 (69.6%)
Q5 Post	10/10 (100%)
Q6 Pre	11/23 (47.8%)
Q6 Post	9/10 (90%)

### Appendix K Post Simulation Participant Survey Results for Site 2

The simulation session today was educational
  (0=not at all educational, 5=the most educational).
  Average score 5, n=6
The simulation session was realistic
  (0=not at all realistic, 5=the most realistic).
  Average score 4.8, n=6
[Author’s] instruction was effective
  (0=not at all effective, 5=the most effective).
  Average score 4.8, n=6

Comments:

- Great Job!
- This was the first time we did a multi sim event so it was really cool. I definitely learned a lot, how to prioritize and also where some of my weakness [sic] lie.
- It would have been helpful to know who was in the room and who was watching but other than that I found the sim to be a great experience.
- I appreciated the first debrief, which allowed us (the participants) an opportunity to review areas that needed improvement so that we could implement them in the second simulation. I look forward to building on this simulation and creating a real outdoor trauma 3, so that learners can get better oriented to the location of the supplies and equipment. Thank you for all your hard work to put this together. Thank the staff who assisted with the simulation.
