# Supplementary figures and images for: Small-Scale High-Fidelity Simulation for Mass Casualty Incident Readiness
Source: J Educ Teach Emerg Med. 2021 Oct 15;6(4):S1–S111. doi: 10.21980/J84S8S (PMC10332734; doi:10.21980/J84S8S)

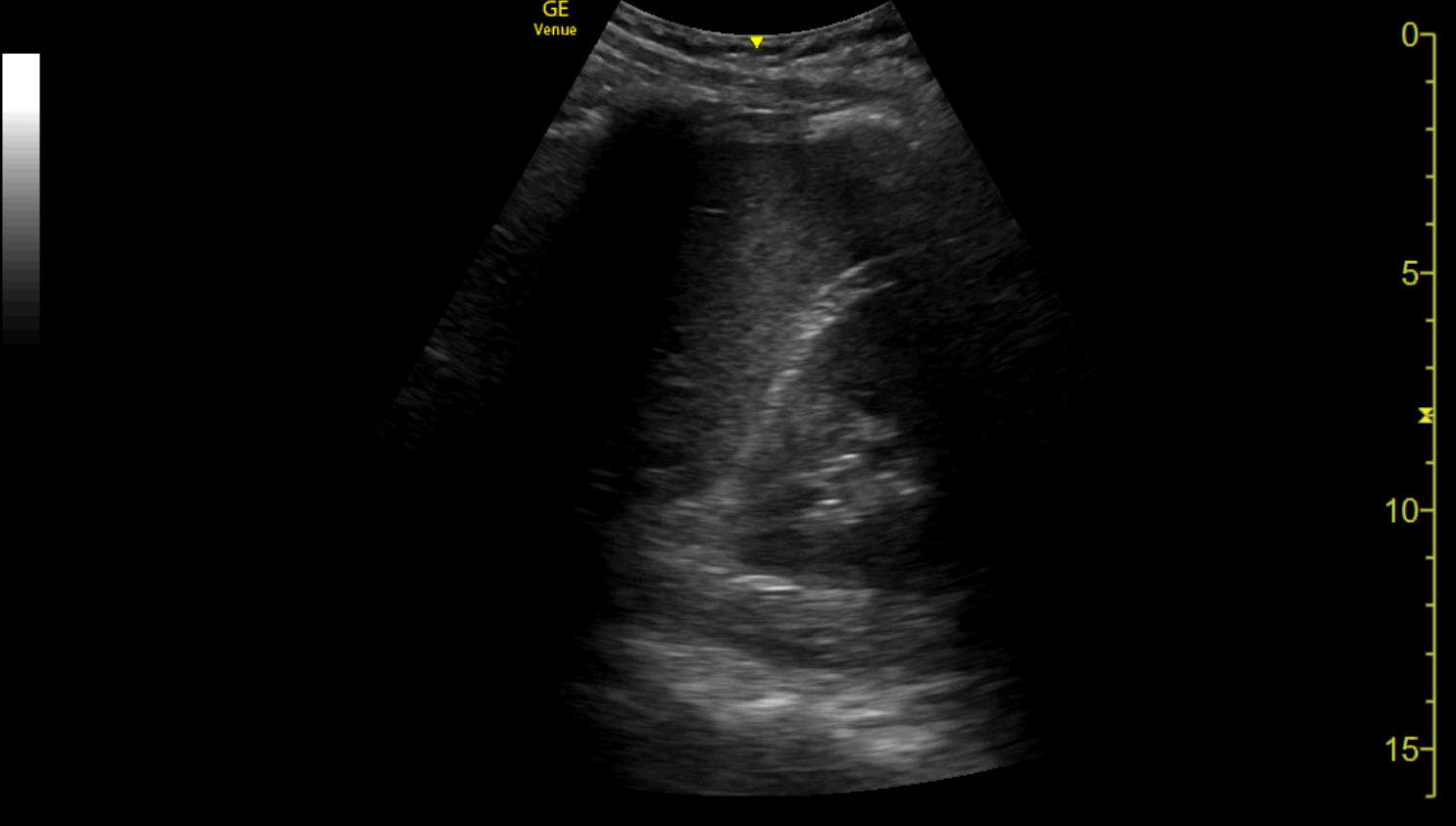

Supplement: Supplementary file 1 [file JETem-6-4-S1-supp1.jpg]

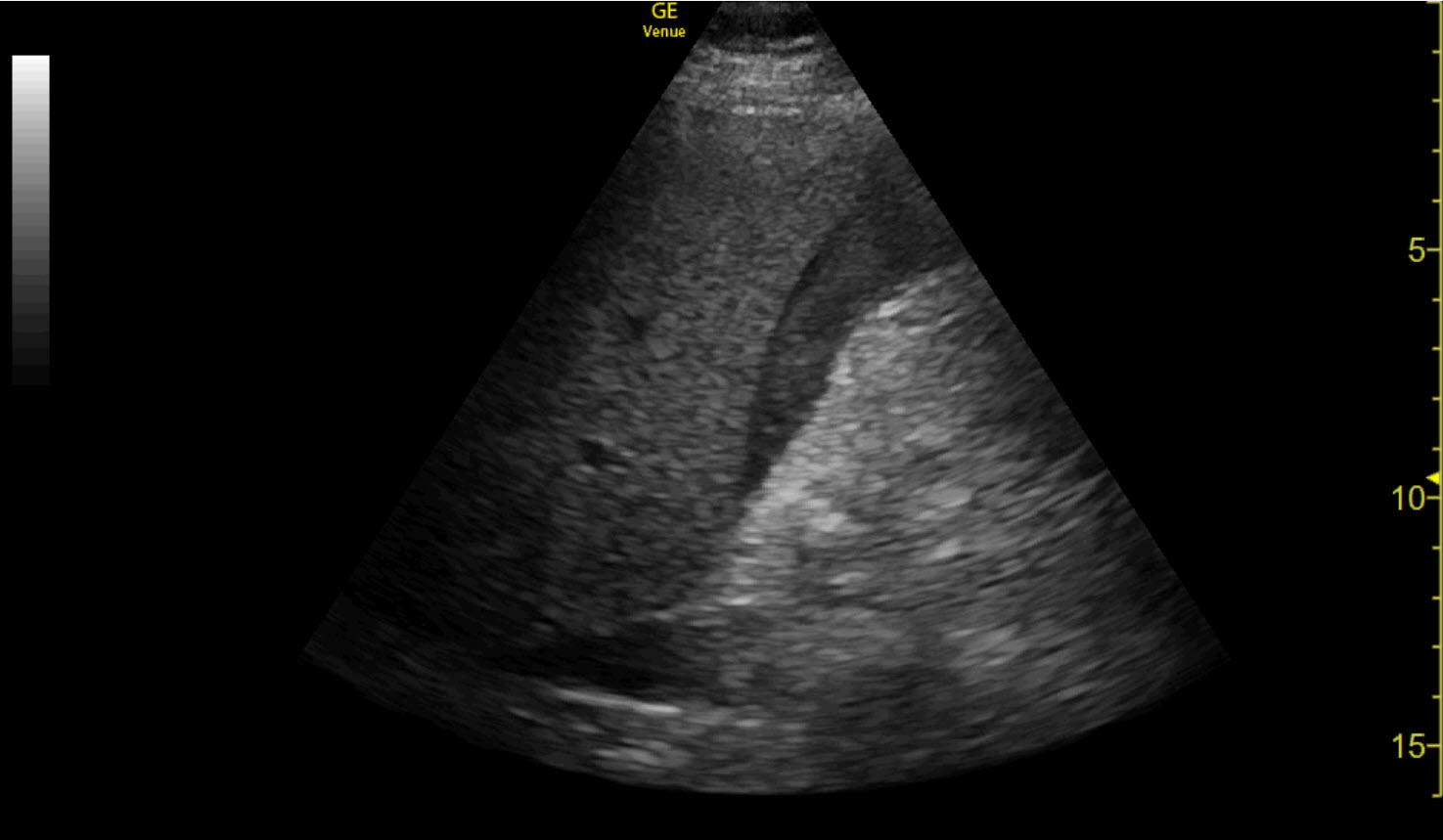

Supplement: Supplementary file 2 [file JETem-6-4-S1-supp2.jpg]

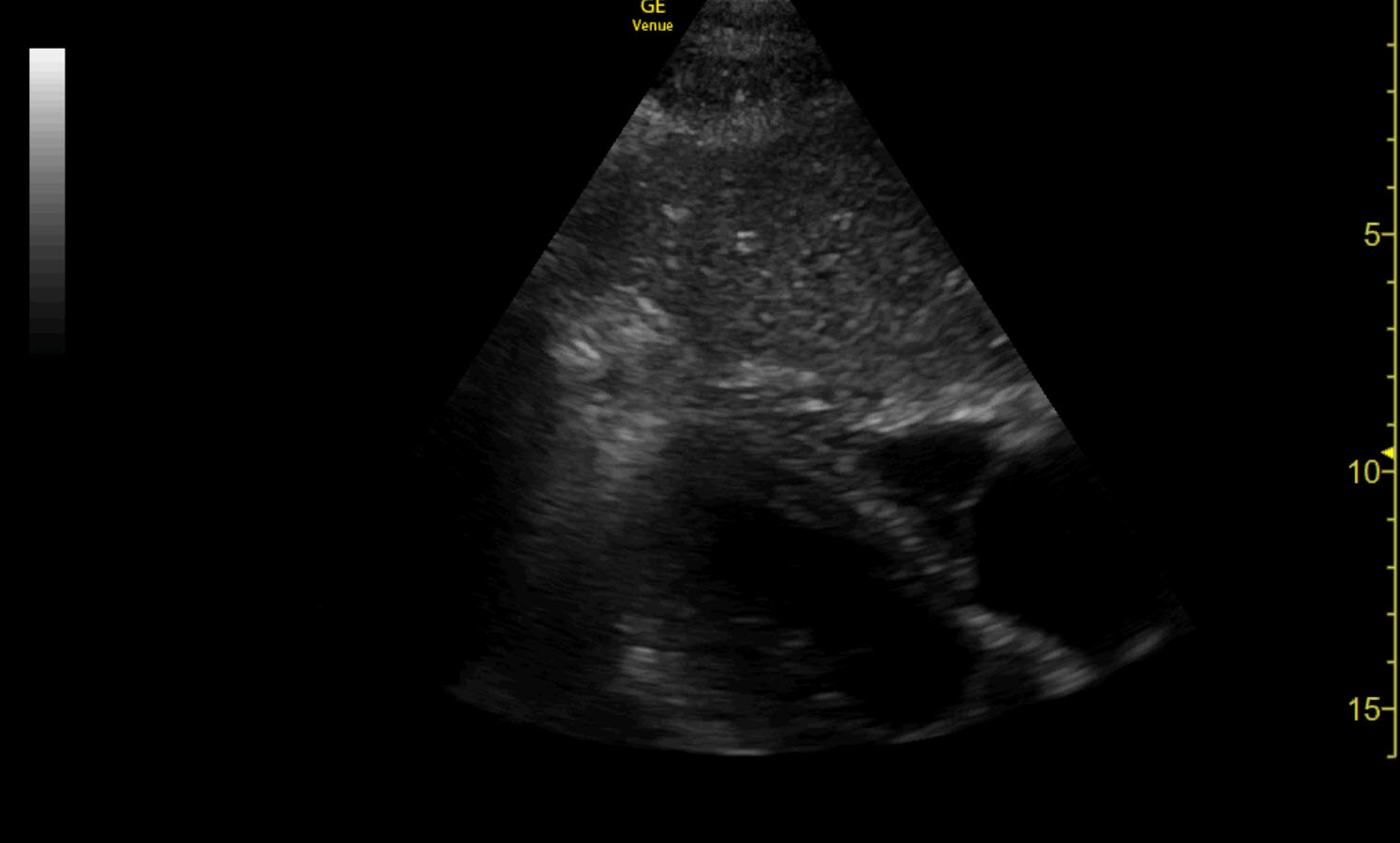

Supplement: Supplementary file 5 [file JETem-6-4-S1-supp5.jpg]
